# Tpz1^TPP1^ prevents telomerase activation and protects telomeres by modulating the Stn1-Ten1 complex in fission yeast

**DOI:** 10.1038/s42003-019-0546-8

**Published:** 2019-08-07

**Authors:** Amanda K. Mennie, Bettina A. Moser, Alice Hoyle, Ross S. Low, Katsunori Tanaka, Toru M. Nakamura

**Affiliations:** 1 Department of Biochemistry and Molecular Genetics, College of Medicine, University of Illinois at Chicago, Chicago, IL 60607 USA; 2 Department of Bioscience, School of Science and Technology, Kwansei Gakuin University, Sanda, 669-1337 Japan; 3 Present Address: Earlham Institute, Norwich Research Park, Norwich, NR4 7UZ United Kingdom

**Keywords:** Telomeres, DNA replication

## Abstract

In both mammalian and fission yeast cells, conserved shelterin and CST (CTC1-STN1-TEN1) complexes play critical roles in protection of telomeres and regulation of telomerase, an enzyme required to overcome the end replication problem. However, molecular details that govern proper coordination among shelterin, CST, and telomerase have not yet been fully understood. Here, we establish a conserved SWSSS motif, located adjacent to the Lys242 SUMOylation site in the fission yeast shelterin subunit Tpz1, as a new functional regulatory element for telomere protection and telomere length homeostasis. The SWSSS motif works redundantly with Lys242 SUMOylation to promote binding of Stn1-Ten1 at telomere and sub-telomere regions to protect against single-strand annealing (SSA)-dependent telomere fusions, and to prevent telomerase accumulation at telomeres. In addition, we provide evidence that the SWSSS motif defines an unanticipated role of Tpz1 in limiting telomerase activation at telomeres to prevent uncontrolled telomere elongation.

## Abstract (web summary)

Amanda Mennie et al. identify a conserved motif in the fission yeast protein Tpz1 that protects telomeres from telomerase accumulation and telomere fusions. They find that the motif acts as a regulatory element synergistically with adjacent SUMOylation to promote binding of Stn1 Ten1 at telomere regions.

## Introduction

In order to stably maintain linear chromosomes, cells have evolved to assemble a protective nucleoprotein complex known as the telomere^1,2^. In most eukaryotic species, telomeric repeat DNA sequences consist of repetitive GT-rich double-stranded DNA (dsDNA) that terminates in a single-stranded DNA (ssDNA) overhang, often referred to as the G-tail^1^. Telomeric repeat extension by telomerase is important to prevent loss of telomeric DNA during successive rounds of DNA replication, due to the “end replication problem” caused by the inability of replicative DNA polymerases to fully replicate ends of linear DNA molecules^1,2^.

The telomeric DNA repeats are bound by a protective telomere complex known as shelterin^3^. Shelterin plays critical roles in preventing telomeres from rearrangement or fusion by DNA repair proteins or causing a permanent cell-cycle arrest mediated by DNA damage and replication checkpoint proteins^4,5^. On the other hand, various DNA damage response and repair proteins, such as Ku, ATM and ATR play critical roles in ensuring telomerase recruitment to telomeres^6–8^. In addition, the highly conserved CST (CTC1/Cdc13-STN1-TEN1) complex contributes to telomere maintenance by collaborating with DNA polymerase α (Polα)-primase to ensure that lagging strand synthesis is completed in a timely manner^9,10^. Mutations that disrupt regulation of shelterin, CST, and/or telomerase complexes have been identified in patients that exhibit signs of premature aging and also in cancer patients, and such findings underscore the importance for better understanding how cells ensure telomere maintenance through proper regulation of these telomere-related complexes^11,12^.

**Fig. 1:**
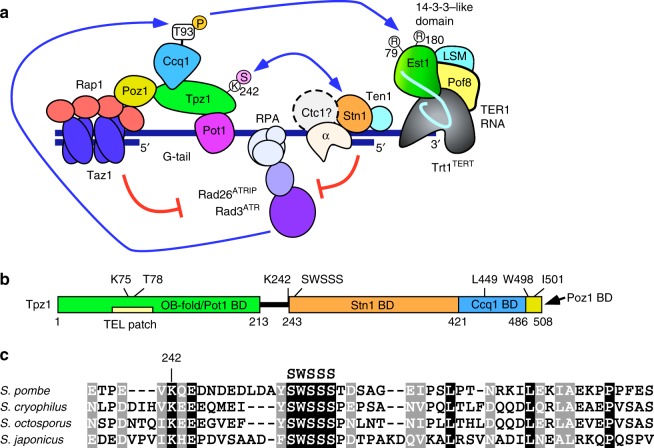
Fission yeast shelterin and Stn1-Ten1 complex. **a** A model depicting regulation of telomerase by shelterin and Stn1-Ten1 complexes in fission yeast via (1) Rad3^ATR^ kinase dependent phosphorylation of Ccq1 Thr93 (yellow circle with “P”) to facilitate telomerase recruitment^7,35^, and (2) Tpz1 Lys242 SUMOylation (pink circle with “S”) to facilitate Stn1-Ten1 recruitment to inhibit telomerase^26,27^. **b** A schematic representation of Tpz1. Protein-protein interaction domains^15,22,28^, residues critical for mediating Tpz1-Ccq1 and Tpz1-Poz1 interactions^22^, TEL patch region^24,25^, predicted OB fold domain^15^, Lys242 SUMOylation site^26,27^, and the SWSSS motif are indicated. **c** Sequence alignment of Tpz1 region critical for its interaction with Stn1-Ten1 in *S. pombe*, and corresponding Tpz1 region from three additional *Schizosaccharomyces* species. Identical residues conserved among all four species are marked black, while amino acid residues that maintain similar chemical properties among all four species are marked gray. Alignment of full length Tpz1 among *Schizosaccharomyces* species is also provided in Supplementary Fig 1

Since fission yeast *Schizosaccharomyces pombe* utilizes both shelterin and Stn1-Ten1 complexes that show good conservation with their mammalian counterparts, it has emerged as an attractive model organism to study telomere regulation. In addition, the ability of fission yeast cells to survive severe telomere dysfunction by circularizing all three chromosomes^13,14^ provides a powerful and convenient experimental assay to monitor loss of telomere function. The fission yeast shelterin complex consists of Taz1, Rap1, Poz1, Tpz1 (ortholog of mammalian TPP1), Ccq1, and Pot1^15^ (Fig. 1a). Taz1 (counterpart of mammalian TRF1 and TRF2 proteins) and Pot1 are telomeric repeat DNA-binding proteins that specifically recognize dsDNA and the ssDNA G-tail, respectively^16,17^. These two DNA-binding proteins are connected by a bridge that is formed by Taz1-Rap1, Rap1-Poz1, Poz1-Tpz1, and Tpz1-Pot1 interactions within shelterin, and these connections appear to play critical roles in both telomere length regulation as well as shelterin-dependent heterochromatin formation at telomeres^18,19^. While it is currently unknown if fission yeast cells utilize a CTC1-like protein (Fig. 1a), conserved Stn1 and Ten1 subunits have been identified^20^. Both shelterin and Stn1-Ten1 complexes are essential for telomere protection, since upon elimination of key components of either shelterin or Stn1-Ten1 (*pot1∆*, *tpz1∆*, *stn1∆*, or *ten1∆*), a majority of cells immediately show rampant telomere fusions and die, and only rare survivors with all circular chromosomes can be recovered^15,16,20^.

Tpz1 serves as an important nexus within the shelterin complex, with additional protein interactions that promote telomerase and Stn1-Ten1 recruitment to telomeres. Tpz1-Ccq1 interaction is essential for generating Rad3^ATR^/Tel1^ATM^-dependent phosphorylation of Ccq1 at Thr93, which promotes interaction between the telomerase regulatory subunit Est1 and Ccq1, and Est1-dependent recruitment of telomerase^21–23^ (Fig. 1a). In addition, evolutionarily conserved “TEL patch” residues within Tpz1 (Fig. 1b) promote telomerase recruitment and activation^24,25^. Furthermore, SUMOylation of Tpz1 at Lys242 promotes telomere binding of the Stn1-Ten1 complex, and restrains telomere elongation by limiting recruitment of telomerase to telomeres^26,27^. However, Tpz1 SUMOylation-dependent localization of Stn1-Ten1 at telomeres appeared to be dispensable for an essential telomere protection function of the Stn1-Ten1 complex, since *tpz1-K242R* cells stably maintain elongated telomeres and do not show telomere fusions^26,27^. In this study, we characterize a novel dual-function regulatory motif within Tpz1 that functions redundantly with Lys242 SUMOylation to promote telomere association of the Stn1-Ten1 complex to protect telomeres, and counteracts TEL-patch-dependent telomerase activation to limit telomere extension.

## Results

### The SWSSS motif in Tpz1 limits telomerase recruitment

**Fig. 2:**
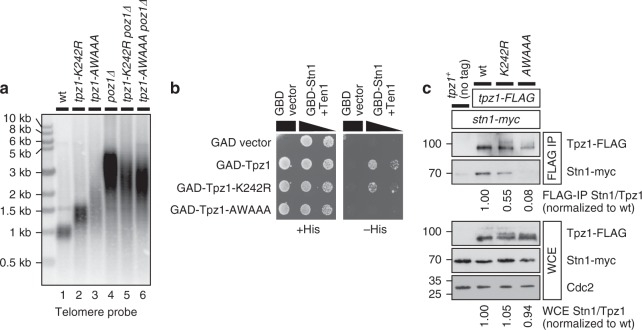
Lys242 SUMOylation and the SWSSS motif contribute to interaction between Tpz1 and Stn1-Ten1. **a** Southern blot analysis of telomere length for indicated mutant strains. All samples were prepared from strains that were extensively restreaked (>150 generations) on YES plates to ensure that terminal telomere lengths were achieved. **b** Y2H assay to monitor interaction between Tpz1 and Stn1-Ten1. The Gal4-activation domain (GAD) was fused to Tpz1, and the Gal4 DNA-binding domain (GBD) was fused to Stn1 and monitored for interaction in the presence of untagged Ten1. Growth on a –His plate indicates interaction. For GBD-Stn1 + Ten1, spots containing 1:5 dilutions are also shown. **c** Examination of Tpz1-Stn1 interaction by co-IP. Cdc2 western blot served as loading control for whole cell extract (WCE). Western blot signals were quantified to determine Stn1/Tpz1 ratio (normalized to wild-type) for FLAG-IP and WCE samples, and shown below blots. Molecular weight (kDa) of size markers are indicated

Adjacent to the Lys242 SUMOylation site, Tpz1 proteins from four *Schizosaccharomyces* species carry a highly conserved “SWSSS” motif (Fig. 1b, c and Supplementary Fig 1). When this motif was mutated to *AWAAA*, cells showed telomere elongation more severe than *tpz1-K242R* cells (Fig. 2a, lanes 2-3). Yeast 2-hybrid (Y2H) and co-IP assays revealed that the *AWAAA* mutation disrupts the interaction between Tpz1 and the Stn1-Ten1 complex more severely than *K242R* (Fig. 2b, c). On the other hand, neither *K242R* nor *AWAAA* affected Tpz1 protein stability or disrupted Tpz1-Poz1, Tpz1-Pot1, or Tpz1-Ccq1 interactions (Supplementary Fig 2).

**Fig. 3:**
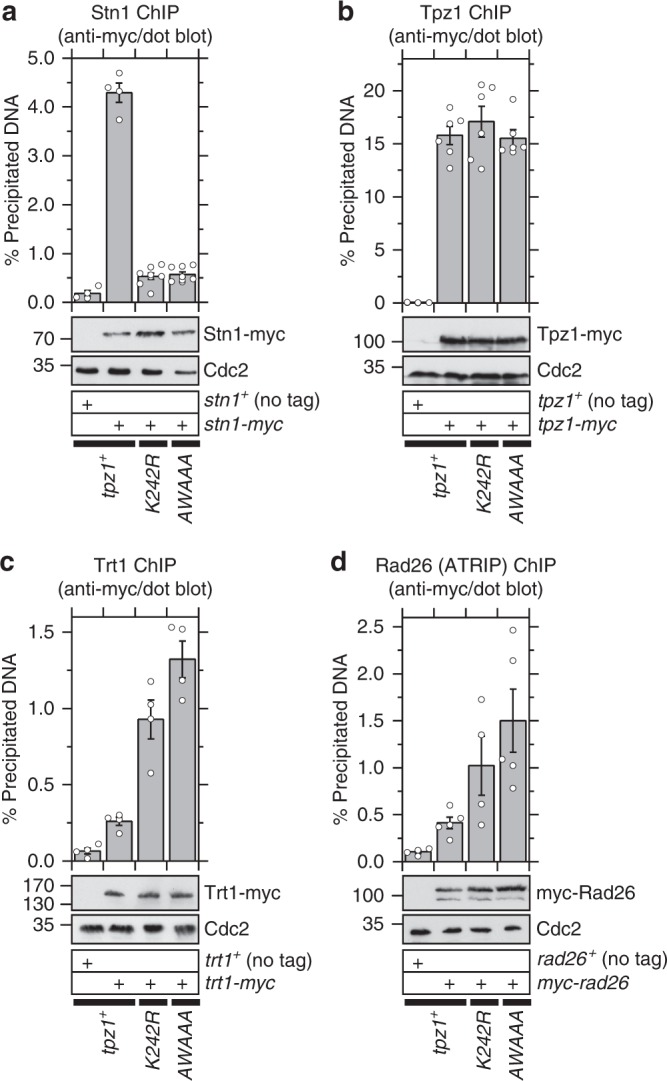
Effects of *K242R* and *AWAAA* mutations on recruitment of various telomere-related factors to telomeres. **a**–**d** Dot-blot ChIP assays from asynchronous cell cultures to monitor binding of **a** Stn1, **b** Tpz1, **c** Trt1^TERT^, and **d** Rad26^ATRIP^ at telomeres. Plots show mean values plus/minus SEM and distribution of individual data points from at least four independent experiments. Raw data values and statistical analysis of ChIP assays by two-tailed Student’s *t*-test are shown in Supplementary Data 1. Expression levels of myc-tagged proteins used in ChIP assays were monitored by western blot analysis. Anti-Cdc2 blots served as loading control. Molecular weight (kDa) of size markers are indicated

Chromatin immunoprecipitation (ChIP) assays found that Stn1 binding to telomeres is greatly reduced in both *K242R* and *AWAAA* mutant cells (Fig. 3a), while association of mutant Tpz1 itself with telomeres is not affected (Fig. 3b). ChIP assays also revealed that *AWAAA* cells show increases in telomere binding for both the telomerase catalytic subunit Trt1^TERT^ and the Rad3^ATR^ kinase regulatory subunit Rad26^ATRIP^ (Fig. 3c, d). Thus, these data revealed a strong correlation between the ability to accumulate Stn1-Ten1 at telomeres through interaction with Tpz1, and the ability to regulate telomere length by limiting Rad3^ATR^-dependent recruitment of telomerase to telomeres.

### SWSSS and K242-SUMO limit Trt1 binding independently of Poz1

**Fig. 4:**
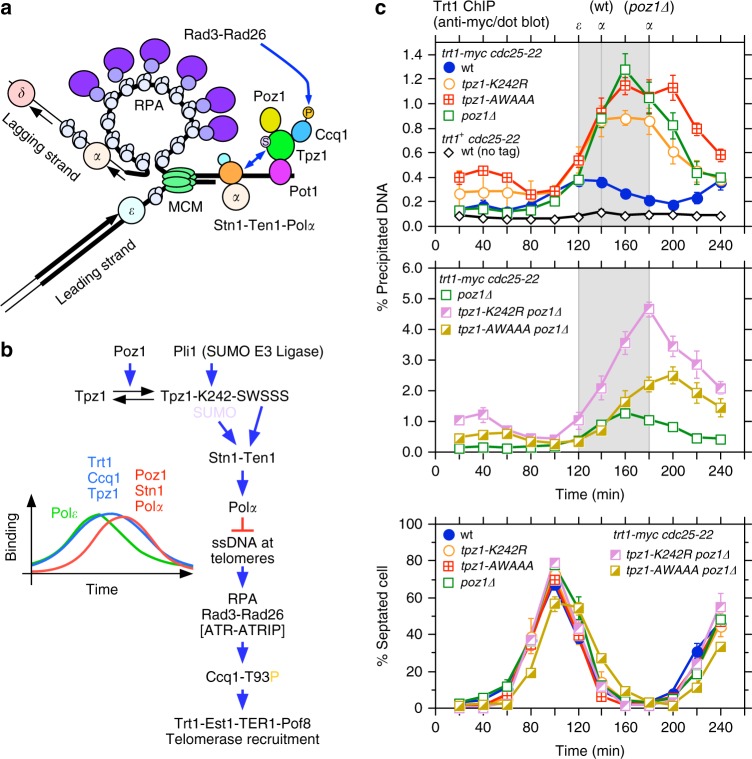
Effects of *K242R* and *AWAAA* mutations on cell-cycle-regulated recruitment of Trt1 at telomeres. **a** A model depicting replicating telomeres in fission yeast^62,63^. **b** Summary of previous cell-cycle-ChIP results for DNA Polymerases, Trt1, Tpz1, Ccq1, Poz1, and Stn1^28^. A proposed model for telomere length regulation pathway^22,28^ is also indicated. **c** Cell-cycle-ChIP assays to monitor telomere binding of Trt1^TERT^. Synchronized cell cultures were obtained using *cdc25-22*, and binding of myc-tagged Trt1 was monitored by dot-blot ChIP assays. Previously determined peaks of binding for leading (Polε) and lagging (Polα) strand DNA polymerases are indicated with gray shaded area^28,63^. DNA Polε binding peaks at 120 min after release from G_2_/M arrest for both wild-type and *poz1∆* cells, while DNA Polα binding peaks at 140 min for wt cells and at 180 min for *poz1∆* cells^28,63^. A plot for % septated cells to monitor cell-cycle progression is also shown. Plots show mean values plus/minus SEM from at least three independent experiments^64^. Raw data values for ChIP assays and % septated cells are shown in Supplementary Data 1

Compared to wild-type cells, *poz1∆* cells carry highly elongated telomeres and exhibit greatly increased and persistent telomerase accumulation in late-S/G_2_, caused by hyper-phosphorylation of Ccq1 Thr93 due to increased accumulation of Replication Protein A (RPA) and Rad3^ATR^-Rad26^ATRIP^ complexes at telomeres^21^. Cell-cycle-ChIP analyses also established that Poz1, Stn1, and Polα show nearly identical temporal telomere binding patterns, and elimination of Poz1 or Rap1 led to substantial and nearly identical delays in recruitment timing for Poz1 (in *rap1∆*), Stn1, and DNA Polα, without affecting the timing of arrival for the leading strand DNA polymerase ε (Polε)^28^ (Fig. 4a, b). Delayed arrival of Stn1 and Polα in *poz1∆* or *rap1∆* cells also correlated with increased and persistent association of telomerase in late S/G_2_-phase^28^. Taking all these observations together, we had thus suggested previously that (1) Tpz1-dependent recruitment of Poz1 to telomeres facilitates the timely recruitment of the Stn1-Ten1 complex and DNA Polα to limit accumulation of ssDNA, RPA, and Rad3^ATR^-Rad26^ATRIP^ at lagging strand telomeres, and that (2) the failure to promote timely lagging strand DNA synthesis is responsible for the substantial increase in Ccq1 Thr93 phosphorylation-dependent telomerase binding at telomeres in late S/G_2_-phases in *poz1∆* or *rap1∆* cells^22,28^ (Fig. 4b).

To better understand why *K242R* and *AWAAA* mutations cause increases in Trt1 binding at telomeres (Fig. 3c), we examined how cell-cycle-regulated Trt1 recruitment is affected in *K242R* and *AWAAA* cells in either *poz1*^*+*^ or *poz1∆* background. We found that, while single mutant strains (*tpz1-K242R*, *tpz1-AWAAA*, and *poz1∆*) show very similar increased and persistent binding of Trt1 in late S/G_2_-phase, double mutant *tpz1-K242R poz1∆* and *tpz1-AWAAA poz1∆* strains show an additional increase in telomere binding of Trt1 (Fig. 4c). Thus, we concluded that Poz1 plays additional role(s) in limiting telomerase accumulation, distinct from either K242 SUMOylation or the SWSSS motif.

On the other hand, Southern blot analysis found that double mutant *tpz1-K242R poz1∆* and *tpz1-AWAAA poz1∆* cells do not show any further telomere elongation but rather show slight telomere shortening compared to *poz1∆* cells (Fig. 2a, lanes 4–6). Previous studies have found that *taz1∆* cells, despite showing a greater increase in telomerase binding at telomeres, show slight telomere shortening compared to *poz1∆* cells due to problems in (1) efficient replication of elongated telomere repeats and (2) protection against exonuclease-dependent degradation of telomeres^29,30^. Thus, Poz1 might play non-overlapping role(s) in regulation of telomere replication and/or protection that is distinct from the Lys242 SUMOylation- and SWSSS motif-dependent regulation of the Stn1-Ten1 complex at telomeres.

### SWSSS and K242-SUMO redundantly protect telomeres

**Fig. 5:**
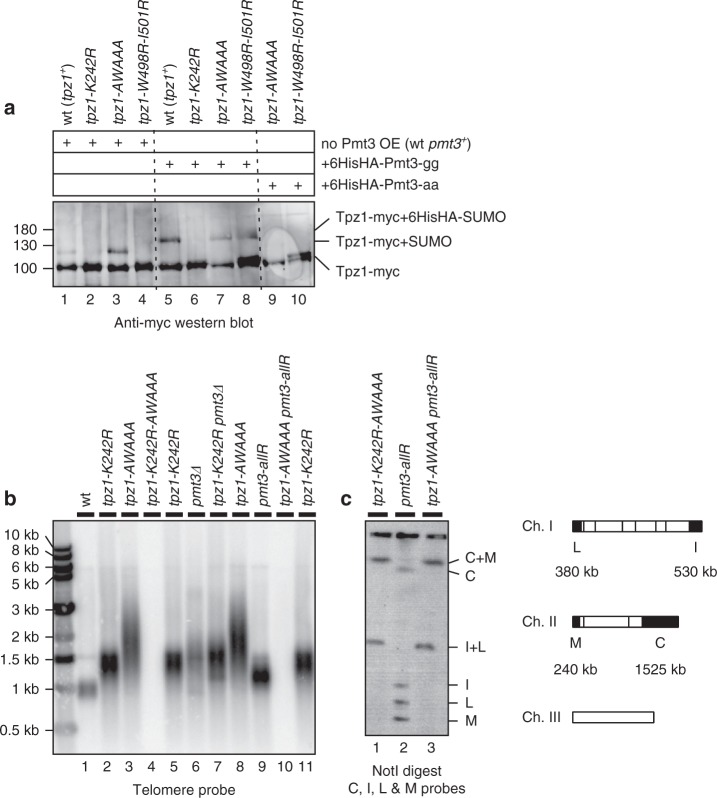
Telomere protection function of Stn1-Ten1 is mediated by Tpz1 Lys242 SUMOylation and the SWSSS motif. **a** Detection of Tpz1 SUMOylation in wild-type and indicated mutant alleles of Tpz1. Denatured whole cell extracts from cells expressing endogenous wild-type Pmt3 only (lanes 1-4), cells ectopically over-expressing conjugatable His6-3HA-tagged Pmt3-gg (lanes 5-8) or non-conjugatable His6-3HA-tagged Pmt3-aa (lanes 9 and 10) from a pREP1-derived vector were processed for anti-myc western blot analysis. Additional western blots and their quantifications to determine changes in Tpz1 SUMOyltion levels are also shown in Supplementary Fig 3. **b** Southern blot analysis of telomere length for indicated mutant strains. For *tpz1-K242R-AWAAA* and *tpz1-AWAAA pmt3-allR* strains, hybridization signals for telomeric repeats were completely lost due to chromosome circularization. **c** Pulsed-field gel analysis of telomeric NotI-fragments. A schematic NotI restriction map of *S. pombe* chromosomes with telomeric NotI fragments (C, I, L, and M) from chromosomes I and II marked as black boxes is also shown

Since *K242R* and *AWAAA* mutations both caused severe reduction in Stn1 localization (Fig. 3a) and similar persistent binding of Trt1 in late S/G_2_-phase (Fig. 4c), we investigated the possibility that the SWSSS motif promotes Stn1 localization at telomeres by promoting Lys242 SUMOylation. However, we found that the *AWAAA* mutation did not reduce but rather increased Lys242 SUMOylation (Fig. 5a, lanes 1 and 3, and Supplementary Fig 3). Intriguingly, *tpz1-W498R-I501R*, which disrupts Tpz1-Poz1 interaction and causes telomere elongation just like *poz1∆*^22^, caused elimination of the signal for the Tpz1 SUMOylation band (Fig. 5a, lane 4, and Supplementary Fig 3a and 3c) when endogenous levels of SUMO (Pmt3) are expressed, suggesting that loss of Poz1 might affect SUMO-dependent regulation of Stn1-Ten1. Over-expression of conjugatable 6His-3HA-Pmt3-gg (but not non-conjugatable 6His-3HA-Pmt3-aa) led to detection of an enhanced and shifted SUMO-Tpz1 band in wild-type, *AWAAA* and *W498R-I501R* cells but not in control *K242R* cells, indicating that neither the SWSSS motif nor Tpz1-Poz1 interaction is essential for Tpz1 Lys 242 SUMOylation (Fig. 5a, lanes 5–8). On the other hand, increase in SUMOylated Tpz1was substantially less in *W498R-I501R* cells compared to wild-type or *AWAAA* cells under SUMO over-expression conditions (Supplementary Fig 3b and 3c), further supporting the notion that Tpz1-Poz1 interaction promotes Tpz1 SUMOylation.

Despite greatly reducing Stn1 binding to telomeres, neither *K242R* nor *AWAAA* mutants shared the immediate telomere de-protection phenotype found in *stn1Δ* or *ten1Δ* cells^20^, where cells exclusively survive by circularizing all chromosomes. Thus, the residual telomere association of Stn1 found in *K242R* and *AWAAA* cells must be sufficient to prevent telomere loss. Since Tpz1 Lys242 SUMOylation is not lost but rather upregulated in *AWAAA* cells, we wondered if Tpz1 SUMOylation might serve as a redundant mechanism to allow *AWAAA* mutant cells to retain partial Stn1-Ten1 function. Indeed, we found that *K242R-AWAAA* double mutant cells immediately lost telomere protection, and survived only as cells carrying circular chromosomes, much like *stn1Δ* and *ten1Δ* cells (Fig. 5b, c). Furthermore, these survivor cells not only lost telomere repeats but also sub-telomere regions (Supplementary Fig 4), much like other well characterized circular chromosome survivor cells in fission yeast (*trt1∆*, *pot1∆*, *tpz1∆*, *stn1∆*, and *ten1∆*)^13,15,16,20^ that circularize their chromosomes via single-strand annealing (SSA) among homology regions found in sub-telomeres^31^ (Supplementary Fig 4c).

Because telomere elongation observed in *pmt3Δ* (SUMO deletion) cells is epistatic to *tpz1-K242R*^26,27^ (Fig. 5b, lanes 5–7), we attempted to investigate if *tpz1-AWAAA pmt3Δ* cells also fail to retain telomeres. However, *tpz1-AWAAA* and *pmt3Δ* showed synthetic lethality when combined. Thus, we tested the genetic interaction between *tpz1-AWAAA* and *pmt3-allR*, where all nine Lysine residues within fission yeast SUMO (Pmt3) were mutated to Arginine and thus unable to form a poly-SUMO chain^32^. We found that *pmt3-allR* itself causes telomere elongation (Fig. 5b, lane 9), and *tpz1-AWAAA pmt3-allR* cells survive exclusively by circularizing their chromosomes (Fig. 5b, lane 10 and 5c), consistent with the notion that the SWSSS motif provides a Lys242 SUMOylation-independent role in allowing Stn1-Ten1 to protect telomeres.

### Development and validation of Tpz1-Stn1 fusion protein

Consistent with the notion that loss of Tpz1 Lys242 SUMOylation-dependent recruitment of the Stn1-Ten1 complex can account for telomere elongation in SUMO deletion mutant (*pmt3∆*) cells, we have previously reported that exogenous over-expression of a Tpz1-Stn1 fusion protein was able to partially suppress telomere elongation in *pmt3∆* cells^26^. However, it remained unclear if the Tpz1-Stn1 fusion protein without over-expression could fully substitute functions of endogenous Tpz1 and Stn1 in telomere regulation.

**Fig. 6:**
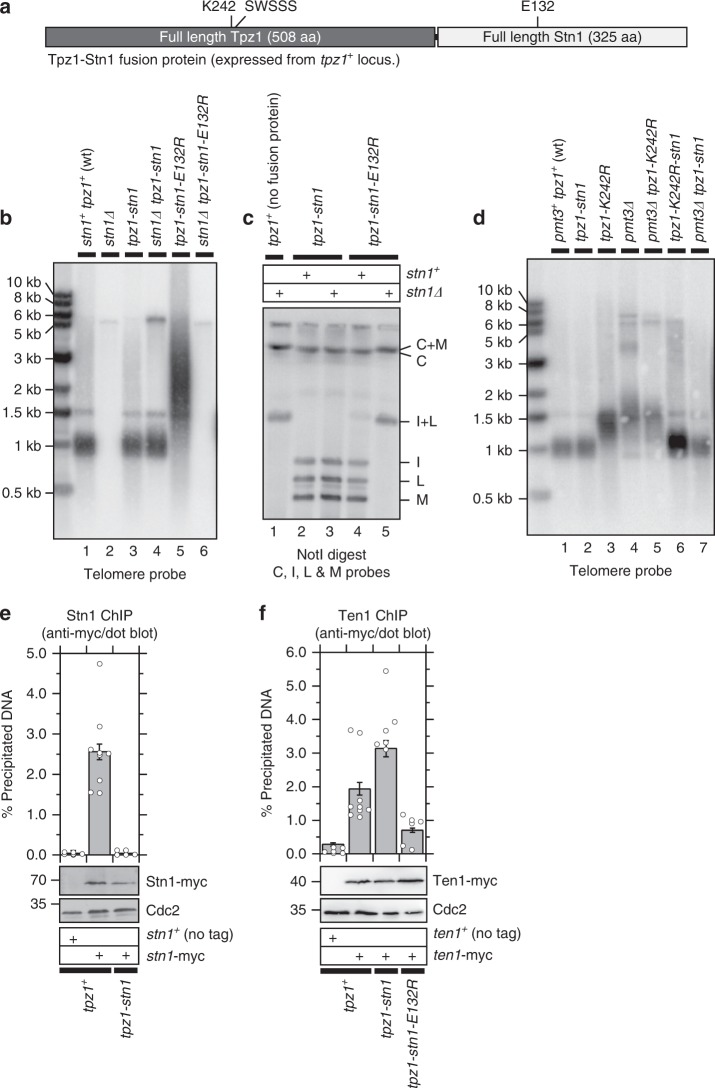
Characterization of strains expressing Tpz1-Stn1 fusion protein. **a** Schematic representation of the Tpz1-Stn1 fusion construct. **b**, **d** Southern blot analysis of telomeres for indicated strains. All samples, except for *stn1∆* and *stn1∆ tpz1-stn1-E132R*, were prepared from strains that were extensively restreaked (>150 generations) on YES plates. For *stn1∆* and *stn1∆ tpz1-stn1-E132R* strains in **b**, hybridization signals for telomeric repeats are completely lost due to chromosome circularization. **c** Pulsed-field gel analysis of telomeric NotI-fragments. **e**, **f** Quantitative dot-blot ChIP assays to monitor telomere localization for **e** Stn1 and **f** Ten1. Protein expression levels for indicated myc-tagged proteins were monitored by western blot, with Cdc2 as a loading control. Molecular weight (kDa) of size markers are indicated. Plots show mean values plus/minus SEM and distribution of individual data points from at least six independent experiments. Raw data values and statistical analysis of ChIP assays by two-tailed Student’s *t*-test are shown in Supplementary Data 1

To better validate the Tpz1-Stn1 fusion protein, we generated strains that express the fusion protein from the *tpz1*^*+*^ locus, controlled by the native *tpz1*^*+*^ promoter (Fig. 6a). Based on western blot analysis, we found that the Tpz1-Stn1 fusion protein is expressed at ~7-fold higher level than endogenous Stn1, but ~5-fold less than native Tpz1 (Supplementary Fig 5a). Remarkably, these cells were able to stably maintain linear telomeres at wild-type length even when the endogenous *stn1* gene was deleted (Fig. 6b, lanes 1–4 and 6c, lanes 1–3), and the Tpz1-Stn1 fusion protein also restored wild-type length telomeres in *tpz1-K242R* or *pmt3∆* cells (Fig. 6d, lanes 6–7).

By contrast, when the *stn1-E132R* mutant allele, which disrupts Stn1-Ten1 interaction in Y2H and co-IP assays (Supplementary Fig 6a-d), is utilized, the resulting fusion protein failed to rescue chromosome circularization in *stn1∆* cells (Fig. 6b, lane 6 and 6c, lane 5), indicating that Stn1 alone is not sufficient for telomere protection but requires its complex partner Ten1. We verified that the inability of Tpz1-Stn1-E132R to rescue *stn1∆* was not due to a loss in shelterin complex formation since co-IP assays found that the Tpz1-Stn1-E132R fusion protein can still interact with Poz1, Pot1, and Ccq1 (Supplementary Fig 6e-g).

Expression of Tpz1-Stn1 strongly interfered with telomere association of endogenous Stn1 (Fig. 6e) while promoting association of Ten1 (Fig. 6f). Conversely, expression of Tpz1-Stn1-E132R caused telomere elongation in *stn1*^*+*^cells (Fig. 6b, lane 5) due to its dominant effect in interfering with localization of endogenous Ten1 (Fig. 6f). These results thus indicated that the Tpz1-Stn1 fusion protein fulfills its telomere function by interacting with endogeneous Ten1, and it can strongly interfere with Tpz1-dependent recruitment of the endogenous Stn1-Ten1 complex to telomeres.

### Targeted Stn1 protects telomeres in *K242R-AWAAA* mutant cells

**Fig. 7:**
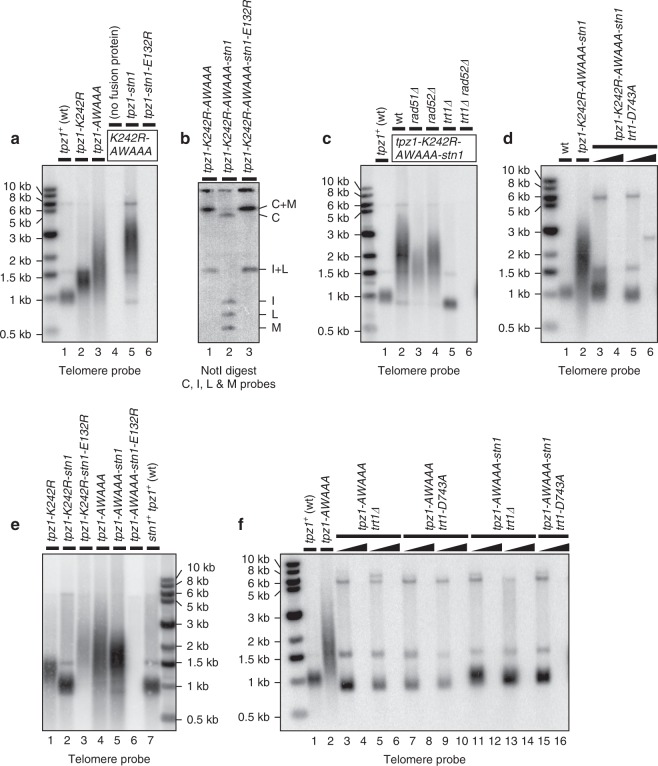
Expression of Tpz1-Stn1 fusion protein can protect telomeres in *tpz1-K242R-AWAAA* cells but fails to restore wild-type telomere length in *tpz1-K242R-AWAAA* or *tpz1-AWAAA* cells. **a** Southern blot analysis of telomere length for indicated mutant strains to test the ability of Tpz1-Stn1 fusion proteins to rescue telomere loss. **b** Pulsed-field gel analysis of telomeric fusions for strains carrying Tpz1-Stn1 fusion proteins. **c** Southern blot analysis to examine requirement of telomerase (Trt1) or HR repair proteins (Rad51 and Rad52) for telomere maintenance in *tpz1-K242R-AWAAA-stn1* cells. **d** Southern blot analysis to examine effect of catalytically dead telomerase (*trt1-D743A*) in *tpz1-K242R-AWAAA-stn1* cells. **e** Southern blot analysis of indicated *tpz1* mutant strains with or without Tpz1-Stn1 fusion protein. **f** Southern blot analysis to test effect of *trt1∆* or *trt1-D743A* for indicated strains. For Southern blots in **a**, **c** and **e**, all samples, except for *tpz1-AWAAA-stn1-E132R*, were prepared from strains that were extensively restreaked (>150 generations) on YES plates. For Southern blots in **d** and **f**, DNA was prepared from early (~30) or late (>150) generations after desired strains were generated by meiotic crosses for *trt1* mutant cells. Right angle triangles were used to signifiy pair of early (left) and late (right) generation for these *trt1* mutant strains

Expression of a Tpz1-K242R-AWAAA-Stn1 fusion protein allowed cells to stably maintain elongated telomeres (Fig. 7a, lane 5 and 7b, lane 2), supporting the notion that failure to recruit the Stn1-Ten1 complex to telomeres is responsible for immediate loss of telomere protection observed in *tpz1-K242R-AWAAA* cells. By contrast, when Stn1-E132R was fused to Tpz1-K242R-AWAAA, it failed to rescue chromosome circularization (Fig. 7a, lane 6 and 7b, lane 3), indicating that forced targeting of Stn1 alone is not sufficient but also requires Ten1 to protect telomeres.

Telomere elongation observed in *tpz1-K242R-AWAAA-stn1* cells is primarily mediated by telomerase since elimination of the telomerase catalytic subunit Trt1 caused telomeres to be stably maintained at slightly shorter than wild-type length in *trt1∆ tpz1-K242R-AWAAA-stn1* cells (Fig. 7c, lane 5). On the other hand, elimination of a homologous recombination (HR) protein, either Rad52 (Rad22) or Rad51 (Rhp51), caused reduction in telomere length (Fig. 7c, lanes 2–4), suggesting that these HR proteins also contribute to telomere extension in *tpz1-K242R-AWAAA-stn1* cells. Consistently, simultaneous elimination of Trt1 and Rad52 led to complete loss of detectable telomeric repeats upon extensive restreaks on plates (Fig. 7c, lane 6). In previous studies, we have shown that catalytically inactive telomerase can prevent HR-based telomere maintenance mechanism^33,34^. Thus, the notion that *tpz1-K242R-AWAAA-stn1* cells utilize both telomerase and HR to maintain elongated telomeres is further supported by the finding that introduction of the catalytically inactive telomerase mutant *trt1-D743A* causes *tpz1-K242R-AWAAA-stn1* cells to completely lose telomeres in later restreaks (Fig. 7d, lanes 4 and 6).

### Tpz1-Stn1 fusion failed to shorten telomeres in *AWAAA* cells

While the Tpz1-Stn1 fusion protein fully restored wild-type telomere length in *tpz1-K242R* or *pmt3∆* cells (Fig. 6d, lanes 6–7 and 7e, lane 2), it was unable to do so in *tpz1-AWAAA* cells (Fig. 7e, lane 5). In addition, expression of Tpz1-AWAAA-Stn1-E132R led to complete loss of telomeres (Fig. 7e, lane 6) even though these cells express wild-type Stn1 and Ten1 proteins. Telomere elongation observed in *tpz1-AWAAA* and *tpz1-AWAAA-stn1* cells is dependent on telomerase, since both *trt1∆* and *trt1-D743A* mutations reversed telomere elongation and caused complete loss of telomeres upon extensive restreaks on plates (Fig. 7f). These observations suggested that the *AWAAA* mutation likely confers additional defect(s) in telomerase regulation that is separable from loss of Stn1-Ten1 recruitment to telomeres. Furthermore, the finding that *trt1∆ tpz1-K242R-AWAAA-stn1* cells stably maintain telomeres but *trt1∆ tpz1-AWAAA-stn1* cells completely lose telomeres (Fig. 7c, lane 5 and 7 f, lanes 12 and 14) indicated that SUMOylation of Lys242 might contribute to preventing HR-based telomere maintenance in *trt1∆ tpz1-AWAAA-stn1* cells.

**Fig. 8:**
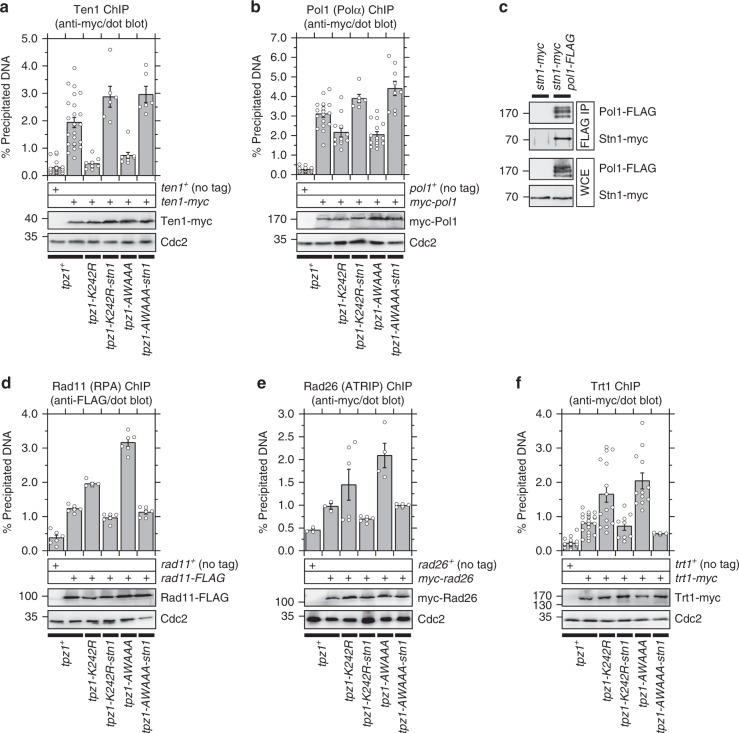
Effects of Tpz1-Stn1 *K242R* and *AWAAA* mutations on recruitment of various telomere-related factors to telomeres. **a**, **b**, **d–f** Dot-blot ChIP assays from asynchronous cell cultures to monitor binding of **a** Ten1, **b** Po11 (DNA Polα), **d** Rad11 (RPA), **e** Rad26^ATRIP^, and **f** Trt1^TERT^ at telomeres. Expression levels of myc- or FLAG-tagged proteins used in ChIP assays were monitored by western blot analysis. Anti-Cdc2 blots served as loading control. Molecular weight (kDa) of size markers are indicated. Plots show mean values plus/minus SEM and distribution of individual data points from at least three independent experiments. Raw data values and statistical analysis of ChIP assays by two-tailed Student’s *t*-test are shown in Supplementary Data 1. **c** Interaction of Stn1 and Po11 (DNA Polα), detected by co-IP analysis

We performed ChIP assays to better understand why the Tpz1-Stn1 fusion protein differs in its abilitiy to restore telomere length in *K242R* vs. *AWAAA* cells. In both *K242R* and *AWAAA* cells, the fusion protein fully restored loss of Ten1 binding (Fig. 8a). Furthermore, while *K242R* and *AWAAA* cells showed reduced DNA Polα binding at telomeres, expression of the fusion protein restored DNA Polα binding to levels greater than wild-type cells for both mutants (Fig. 8b). Previous cell-cycle-ChIP analysis found that arrival of DNA Polα at telomeres coincides with an increase in Stn1 binding^28^ (Fig. 4b). In addition, we detected robust interaction between Stn1 and DNA Polα by co-IP (Fig. 8c). Thus, these data indicated that (1) the Stn1-Ten1 complex promotes recruitment of DNA Polα to telomeres in fission yeast, and (2) failture of the Tpz1-Stn1 fusion protein to reduce telomere length in *AWAAA* cells is not due to failure of the fusion protein to restore binding of Ten1 or DNA Polα.

Expression of Tpz1-Stn1 also reduced telomere association of the RPA subunit Rad11, as well as Rad26^ATRIP^ to levels comparable to wild-type *tpz1*^*+*^ cells for both *K242R* and *AWAAA* (Fig. 8d, e). This is consistent with the idea that the fusion protein, by restoring Ten1 and DNA Polα binding at telomeres, promotes DNA lagging strand synthesis to limit accumulation of ssDNA at telomeres (Fig. 4b). In fact, ChIP analysis of sub-telomere regions found that *K242R* and *AWAAA* mutations also cause reduction in Ten1 binding, alongside increases in Rad11 and Rad26 binding, as far as ~17 kb from the ends of chromosomes, and that the fusion protein is effective in reversing these changes at sub-telomere regions in both mutant backgrounds (Supplementary Fig 7).

Previous studies have established that Rad3^ATR^-Rad26^ATRIP^-dependent phoshorylation of Ccq1 Thr93 promotes telomerase recruitment to telomeres^21,35^. Thus, based on the ability of Tpz1-Stn1 fusion protein to reduce Rad26^ATRIP^ binding, we expected that expression of Tpz1-Stn1 would reduce telomerase binding at telomeres. Indeed, we found that Trt1 binding at telomeres is reduced to levels equivalent or even slightly lower than wild-type *tpz1*^*+*^ cells in both *K242R* and *AWAAA* Tpz1-Stn1 fusion protein backgrounds (Fig. 8f). Thus, we can exclude the possibilty that the Tpz1-Stn1 fusion protein is somehow less functional in limiting Trt1 binding at telomeres in the context of *AWAAA* mutation than in *K242R* mutation.

### The SWSSS motif counteracts telomerase activation

**Fig. 9:**
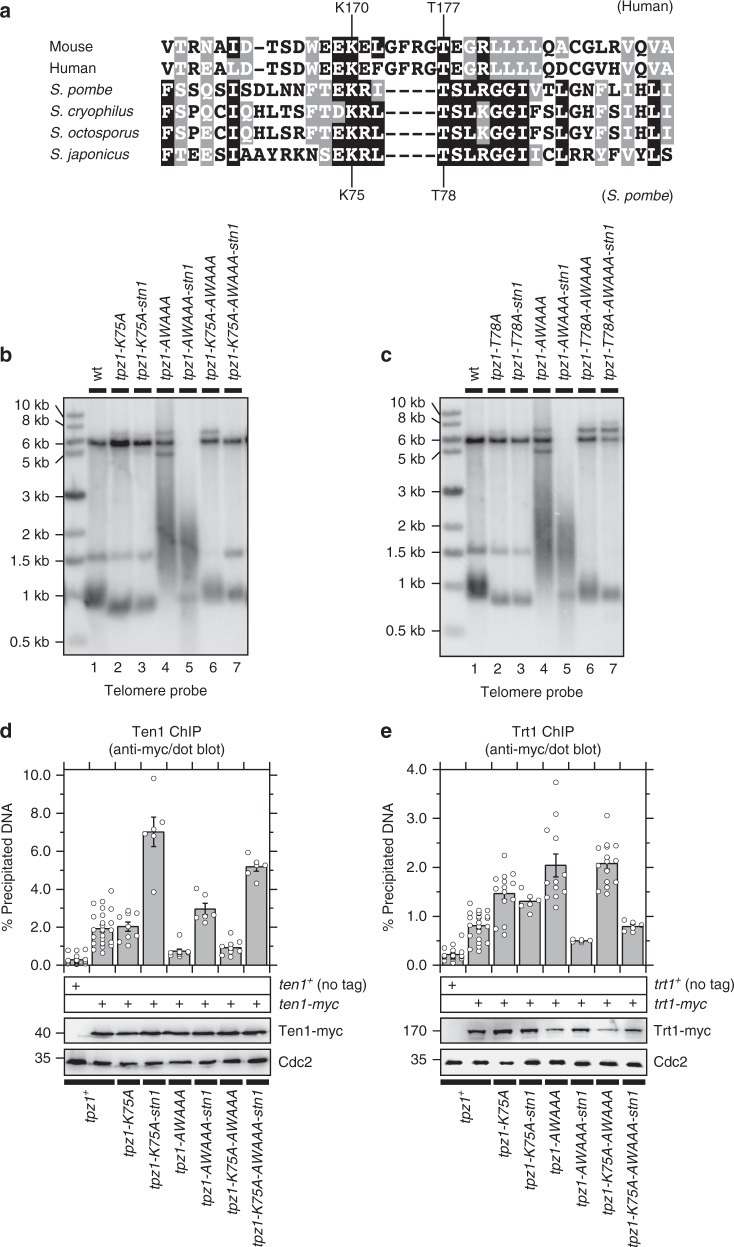
Effects of TEL-patch mutants in *tpz1-AWAAA* mutant. **a** Sequence alignment of the TEL-patch region of TPP1 from human and mouse and Tpz1 from four *Schizosaccharomyces* species. Identical residues conserved among four or greater species are marked black, while amino acid residues that maintain similar chemical properties are marked gray. TEL-patch residues in Tpz1 mutated in this study and potentially equivalent sites in human TPP1 are also indicated. **b**, **c** Southern blot analysis to test effect of **b** *K75A* or **c** *T78A* in *AWAAA* mutants with or without Tpz1-Stn1 fusion on telomere length. All genomic DNA samples were prepared from cells that have been extensively restreaked (>150 generations) on YES plates. While strains used in Southern blot analysis did not carry an epitope tag, corresponding myc-tagged versions of wild-type and mutants (with or without Stn1 fusion) were used in western blot and co-IP experiments, and results indicated that none of the mutant combinations greatly affected protein expression or disrupted interaction of Tpz1 or Tpz1-Stn1 to Poz1, Pot1 or Ccq1 (Supplementary Fig 5b and 8). **d**, **e** Dot-blot ChIP assays from asynchronous cell cultures to monitor binding of **d** Ten1 and **e** Trt1^TERT^ at telomeres. Expression levels of myc-tagged proteins used in ChIP assays were monitored by western blot analysis. Anti-Cdc2 blots served as loading control. Molecular weight (kDa) of size markers are indicated. Plots show mean values plus/minus SEM and distribution of individual data points from at least six independent experiments. Raw data values and statistical analysis of ChIP assays by two-tailed Student’s *t*-test are shown in Supplementary Data 1

Since *tpz1-AWAAA-stn1* cells show elongated telomeres despite reduced Trt1 binding compared to wild-type cells, it appears as if telomerase is more active for telomere extension in *tpz1-AWAAA-stn1* cells. A previous study in fission yeast has implicated an evolutionarily conserved “TEL-patch” region within Tpz1 in activation of telomerase, since mutations in this region (*K75A* and *T78A*) (Figs. 1b, 9a) caused substantial telomere shortening even though telomere association of telomerase was actually increased^24^. Thus, we decided to test the possibility that the SWSSS motif might be involved in restraining TEL-patch-dependent activation of telomerase extension.

We found that introduction of either *K75A* or *T78A* reversed telomere elongation observed in both *tpz1-AWAAA* and *tpz1-AWAAA-stn1* cells, consistent with the notion that telomere elongation observed in these cells is indeed dependent on TEL-patch-dependent telomerase activation (Fig. 9b, c, lanes 4–7). Closer examination revealed that *tpz1-K75A-AWAAA* and *tpz1-T78A-AWAAA* cells show telomere length similar to wild-type cells, but introduction of the fusion construct into these mutants reduced telomere length (Fig. 9b, c, lanes 6–7). ChIP assays found that *tpz1-AWAAA* and *tpz1-K75A-AWAAA* cells show comparable reduction in Ten1 binding (Fig. 9d), and a substantial increase in Trt1 binding that is greater than observed in the *K75A* mutant alone (Fig. 9e). On the other hand, expression of the Tpz1-K75A-AWAAA-Stn1 fusion protein restored telomere association of Ten1 (Fig. 9d), and reduced telomere association of Trt1 (Fig. 9e).

These results indicated that, while the *tpz1-AWAAA* mutation can still reverse telomere shortening in TEL-patch mutant cells through increased accumulation of Trt1, telomere length once again becomes sensitive to the amount of telomerase recruited to telomeres, since a reduction in Trt1 binding imposed by the Tpz1-Stn1 fusion protein is sufficient to limit telomere elongation in *tpz1-K75A-AWAAA* and *tpz1-T78A-AWAAA* cells. Therefore, we concluded that the SWSSS motif of Tpz1 plays an unanticipated function in preventing telomerase activation to limit telomere extension by telomerase, which is functionally separable from the SWSSS motif’s role in promoting Stn1-Ten1 localization at telomeres to limit telomerase recruitment and to protect telomeres against telomere fusions.

## Discussion

**Fig. 10:**
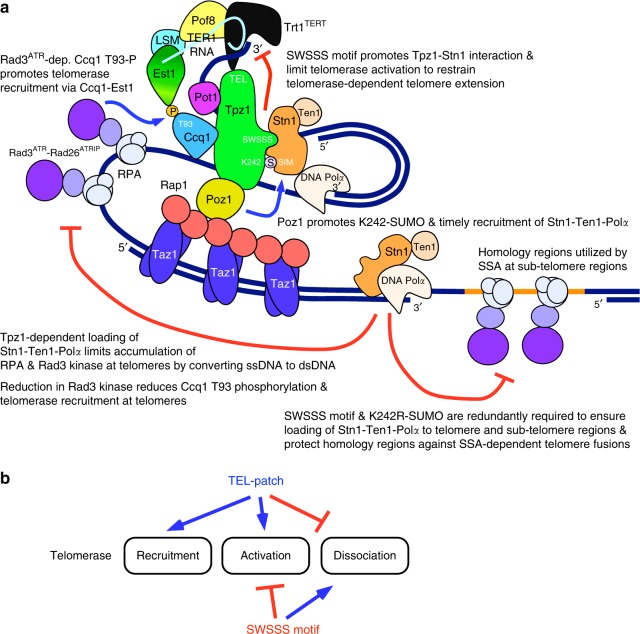
Summary and working model for telomere regulation. **a** A working model depicting how the SWSSS motif contributes to promote Stn1-Ten1-dependent telomere protection and prevent telomere extension by coordinating with Ccq1 Thr93 phosphorylation and Tpz1 Lys242 SUMOylation. **b** A summary of how TEL-patch and SWSSS motifs contribute to telomere length regulation by modulating telomerase recruitment, activation, and dissociation at telomeres

In this study, we investigated the mechanism by which shelterin and Stn1-Ten1 collaborate to ensure stable maintenance of telomeres. We showed that the conserved SWSSS motif within Tpz1 promotes interaction between Stn1 and Tpz1 to facilitate Stn1-Ten1 complex binding to telomeres (Figs. 2, 3), and limits accumulation of telomerase at telomeres in late-S/G_2_ phase (Fig. 4). Since Tpz1 Lys242 SUMOylation is also cell-cycle-regulated and occurs primarily in late S/G_2_-phase^26,27^, we suggest that the SWSSS motif and Lys242 SUMOylation collaborate in late S/G_2_ to achieve optimal recruitment of the Stn1-Ten1 complex to protect telomeres against DNA damage response factors and to limit accumulation of telomerase (Fig. 10a). The finding that the Tpz1-Poz1 interaction-disruption mutant *tpz1-W498R-I501R* shows reduced Lys242 SUMOylation (Fig. 5a) could also explain why *poz1∆* cells show delayed recruitment of Stn1-Ten1-Polα at telomeres^28^, and why *poz1∆*, *tpz1-K242R* and *tpz1-AWAAA* mutant cells show similar persistent accumulation of Trt1^TERT^ in G_2_-phase (Fig. 4c).

We also found that simultaneous loss of the SWSSS motif and Tpz1 Lys242 SUMOylation results in complete loss of telomere protection, much like the phenotype observed in *stn1∆* or *ten1∆* cells (Fig. 5). Importantly, restoration of Stn1-Ten1 localization to telomeres by introduction of the Tpz1-Stn1 fusion protein rescued the loss of telomere protection found in *tpz1-K242R-AWAAA* cells (Fig. 7). Thus, the interaction between Stn1-Ten1 and Tpz1 is promoted by at least two distinct protein-protein interaction regions, one that recognizes SUMOylated Lys242 and another that recognizes the region surrounding the SWSSS motif (Fig. 10a). Our results further imply that the fission yeast Stn1-Ten1 complex is completely dependent on Tpz1-Stn1 interaction for its telomere functions.

In fact, ChIP analysis of sub-telomere regions revealed that Tpz1 plays a critical role in limiting the accumulation of ssDNA not only at telomeres but also at sub-telomere regions as far as ~17 kb from the telomeric end (Supplementary Fig 7). Recent studies have also found that the Stn1-Ten1 complex contributes not just to the replication of telomere repeats but also sub-telomere regions in fission yeast^36,37^. Thus, we suggest that *tpz1-K242R-AWAAA*, *stn1∆*, and *ten1∆* mutant cells all lose telomere protection due to the inability of these mutant cells to complete lagging strand synthesis in a timely manner, which triggers chromosome fusions and loss of distal sequences in the sub-telomere homology regions through a SSA mechanism^31^ (Supplementary Fig. 4c). Conversely, partial retention in Stn1-Ten1 and DNA Polα functions in *K242R* and *AWAAA* single mutant cells must be sufficient to protect against SSA-dependent fusions, but not enough to limit increases in telomerase binding due to increased Rad3^ATR^-dependent Ccq1 Thr93 phosphorylation^21,35^ (Fig. 10a).

A recent study has shown that the C-terminal domain of Stn1, which encompasses tandem winged-helix-turn-helix (WH) domains, is responsible for interaction with Tpz1, and that a SUMO-Interacting Motif (SIM) ILAL sequence located within the 1st WH domain (amino acid residues 226–229) is required to recognize Tpz1 Lys242 SUMO^37^ (Supplementary Fig. 6a). However, observed phenotypes for a *stn1* SIM mutant allele (*stn1-226*) was much more severe than for *tpz1-K242R*, as only *stn1-226* cells showed temperature sensitivity and lost telomere and sub-telomere regions upon continued growth at non-permissive temperature^37^. Intriguingly, *stn1-226 tpz1-K242R* double mutant cells were found to completely lose telomeres even at permissive temperature^37^, similar to the finding for *tpz1-K242R-AWAAA* cells reported in this study. Thus, *stn1-226* cells appear to be paradoxically dependent on Tpz1 Lys242 SUMOylation. One possible interpretation is that the Stn1 region that recognizes and interacts with the SWSSS motif is in close proximity with the SIM, and as such, *stn1-226* mutation might affect the ability of Stn1 to recognize the SWSSS motif region of Tpz1. It is also worth noting that fission yeast Stn1 contains a putative second SIM motif IIYL (amino acid residues 195–198)^37^, which might allow the Stn1-226 mutant protein to still recognize Tpz1 Lys242 SUMOylation (Supplementary Fig. 6a).

Characterization of the Tpz1-Stn1 fusion protein in *tpz1-AWAAA* cells also uncovered an unanticipated role of the SWSSS motif in limiting telomere extension activity of telomerase at telomeres, which is functionally separable from the role of the SWSSS motif in promoting telomere association of the Stn1-Ten1 complex to limit accumulation of RPA, Rad3^ATR^ kinase, and telomerase (Figs. 8, 9). The additional requirement of the SWSSS motif in restraining telomerase extension also explains why *tpz1-K242R-AWAAA-stn1* cells show highly elongated telomeres (Fig. 7a). Thus, we propose that direct Tpz1-Stn1 interaction, strongly mediated by the SWSSS motif, is required for the Stn1-Ten1 complex to limit telomerase activation function (Fig. 10a), although it is formally possible that the SWSSS motif itself, independently of its role in mediating Tpz1-Stn1 interaction, might directly affect telomerase activation at telomeres. We note that the proposed dual functions of the SWSSS motif in limiting telomerase activation and promoting telomerase dissociation are mirror image of previously determined functions of the TEL-patch residues in promoting telomerase recruitment and activation^38–40^, and in preventing premature dissociation of telomerase from telomeres^41^ (Fig. 10b). It would thus be of interest for future studies to determine exactly how these opposing regulatory forces are coordinated to achieve optimal regulation of telomerase action at telomeres.

While previous studies have implicated CST in negative regulation of telomerase-dependent telomere extension in both mammalian and budding yeast cells, general conclusions from these studies have been that the CST-dependent regulation of the DNA Pol α-primase complex is responsible for limiting telomere extension by telomerase^42–45^. On the other hand, mammalian shelterin has also been found to interact with the CST complex^43,46^, and mutations and depletion of Pot1 affect CST-dependent lagging strand synthesis at telomeres^47–49^. Furthermore, an in vitro study of human telomerase has found that addition of the CST complex can prevent POT1-TPP1-dependent stimulation of telomere repeat addition processivity of telomerase, known to depend on direct interaction between the TPP1 TEL-patch and the TEN-domain of the telomerase catalytic subunit TERT^40,43^. Thus, it is possible that a direct interaction between shelterin and CST could also play important roles not only in CST-dependent lagging strand synthesis, but also in negative regulation of the TEL-patch-dependent telomerase activation in mammalian cells.

Budding yeast cells lack a canonical shelterin complex, and instead utilize Cdc13 as a central hub that serves dual roles in recruitment and regulation of both telomerase and Stn1-Ten1-Polα activities^42,50–52^, with Cdc13-Stn1 interaction promoted by SUMOylation of Cdc13, much like Tpz1-Stn1 interaction^52^. Interestingly, budding yeast *stn1* mutations within the C-terminal tandem WH domains have been found to cause telomere elongation, and more active telomerase, based on de novo telomere formation assays^53^. Thus, Stn1 WH domains in budding yeast could also be responsible for modulating telomerase activity by regulating TEL-patch residues found on telomerase subunit Est3 (a potential counterpart of TPP1/Tpz1 in budding yeast). Moreover, it is also worth noting that *Tetrahymena* cells utilize the telomerase subunit p50 (also a potential counterpart of Tpz1/TPP1) that serves dual roles in recruitment and activation of telomerase, and directly interacts with the CST (p75-p45-p19) complex^54–57^. Thus, the dynamic and intimate co-regulation of telomerase and CST by the TPP1-like protein may be a universal feature that is essential for telomere protection among all eukaryotic cells that utilize telomerase to maintain stable linear chromosome ends.

## Methods

### Yeast strains, plasmids, and primers

Fission yeast strains used in this study were constructed using standard methods^58^. Fission yeast cells were cultured in YES (Yeast Extract with Supplements) media^58^ at 32 °C for most experiments, except for those involving *cdc25-22* cells, which were cultured at 25 °C. To combine various mutations or epitope tagged alleles in fission yeast, genetic crosses between opposite mating type strains were carried out in ME (Malt Extract) media^58^ at either 25 or 32 °C, and meiotic haploid off-springs were separated by using a Zeiss dissection microscope. Fission yeast strain genotypes are listed in Table 1 of Supplementary Data 2. Additional details regarding newly generated strains or sources of various deletions and tagged fission yeast strains are listed in Table 2 of Supplementary Data 2. Plasmids used in this study are listed in Table 3 of Supplementary Data 2. Various point mutation and truncation constructs were generated with Phusion (NEB), Q5 (NEB), or QuikChange Lightning (Agilent) site-directed mutagenesis kits. DNA primers used in this study are listed in Table 4 of Supplementary Data 2.

### Yeast two-hybrid assay

Yeast two-hybrid assays were performed by mating *S. cerevisiae MAT*a (Y2HGold: *MAT*a *trp1-901 leu2-3,-112 ura3-52 his3-200 LYS2::GAL1(UAS)-GAL1(TATA)-HIS3 GAL2(UAS)-GAL2(TATA)-ADE2 gal4Δ gal80Δ URA3::MEL1(UAS)-MEL1(TATA)-AUR1-C MEL1*) strains harboring GAL4-DBD (DNA-binding domain) plasmids with *MAT*α (Y187: *MAT*α *trp1-901 leu2-3,-112 ura3-52 his3-200 ade2-101 gal4Δ gal80Δ met*^*-*^ *URA3::GAL1(UAS)-GAL1(TATA)-LacZ MEL1*) strains harboring GAL4-AD (activation domain) plasmids by following instructions in the MATCHMAKER system manual (Clontech)^22^. Positive two-hybrid interactions were identified by spotting mated cells onto SD−HTL (−His) or SD−HTLA (−His −Ade) plates.

### Pulsed-field gel electrophoresis and Southern blot analysis

To prepare agarose plugs containing chromosomal DNA, cells were first washed in SP1 (50 mM citrate-phosphate [pH 5.6], 40 mM EDTA, 1.2 M sorbitol) and then incubated for ~1 h at 37 °C in SP1 with 0.6 mg per ml Zymolyase-100T (ICN Biomedicals). Cells were then pelleted and resuspended at 6–7 × 10^8^ cells per ml in TSE (10 mM Tris-HCl [pH 7.5], 0.9 M sorbitol, 45 mM EDTA). The cell suspension was warmed to 42 °C, and 1 volume of 1.1% low-melting agarose (BioRad) in TSE was added. Aliquots were dispensed into plug molds and allowed to solidify, and plugs were then incubated at 50 °C for 2 h in 0.25 M EDTA, 50 mM Tris-HCl (pH 7.5), 1% SDS and then 48 h in 1% lauryl sarcosine, 0.5 M EDTA, 10 mM Tris (pH 9.5), 1 mg per ml proteinase K. Plugs were washed 4× in T10 × E buffer (10 mM Tris [pH 7.5], 10 mM EDTA) and stored at 4 °C in T10xE buffer. NotI-digested chromosomal DNAs embedded in agarose plugs were separated on 1% agarose gel with 0.5 × TBE buffer at 14 °C, using the BioRad CHEF-DR III system at 6 V cm^−1^ (200 V) and a pulse time of 60–120 s for 24 h. ^32^P labeled probes specific for telomeric NotI fragments from chromosomes I and II (C, I, L, and M fragments as indicated in Fig. 3c) were used to analyze telomeric Not I fragments by Southern blot analysis^13,59^.

### Southern blot analysis

Genomic DNA was prepared by resuspending cell pellets in equal volumes of DNA buffer (100 mM Tris pH 8.0, 1 mM EDTA pH 8.0, 100 mM NaCl, 1% SDS) and Phenol:chloroform:isoamyl alcohol (25:24:1) saturated with 10 mM Tris (pH 8.0) and 1 mM EDTA, and lysing cells with glass beads using FastPrep (MP Biomedicals). Eight hundred nanogram genomic DNA was digested overnight at 37 °C, separated on 1% agarose gel, transferred to Hybond-XL (GE Healthcare), probed with ^32^P labeled telomeric DNA probe, and visualized with Amersham Typhoon Phosphorimager (GE Healthcare)^34,59^.

### Co-immunoprecipitation and western blot analysis

Cells were lysed in lysis buffer (50 mM Tris pH 8.0, 150 mM NaCl, 10% glycerol, 5 mM EDTA pH 8.0, 0.5% NP-40, 50 mM NaF, 1 mM DTT, 1 mM Na_3_VO_4_, 1 mM PMSF, Roche complete protease inhibitor cocktail), and lysed with glass beads with FastPrep (MP Biomedicals)^7^. For Tpz1-Stn1 co-IP, 2 mM DSP (Fisher Scientific PI22585) was added to stabilize the interaction. For analysis of Tpz1 SUMOylation, denaturing extract was prepared^26,60^, and processed for western blot analysis. Proteins were immunoprecipitated using Dynabeads Protein G (Thermo Fisher) and either monoclonal anti-myc antibody (9B11, Cell Signaling), monoclonal anti-FLAG (M2 F1804, Sigma), or monoclonal anti-GFP (clones 7.1 and 13.1, Roche). Western blot analysis was performed using monoclonal anti-FLAG (M2 F1804) at 1:3000, monoclonal anti-myc (9B11) at 1:12,000, monoclonal anti-GFP (clones 7.1 and 13.1) at 1:2000, monoclonal anti-Cdc2 (y100.4, SCBT sc-53217) at 1:5000, or monoclonal anti-α-tubulin (clone B-5-1-2 T5168, Sigma) at 1:5000 dilutions as primary antibodies. HRP-conjugated (goat) anti-mouse (31430, Pierce) was used as the secondary antibody at 1:5000 dilution. LI-COR Image Studio software was used to quantify western blots. Uncropped images of all western blots are shown in Supplementary Fig 9.

### ChIP assay

Exponentially growing cells were crosslinked with formaldehyde by addition of 1/10 volume of fixation solution (11% formaldehyde, 100 mM NaCl, 1 mM EDTA pH 8.0, 0.5 mM EGTA pH 8.0, 50 mM Tris-HCl pH 8.0) for 20 min at room temperature, incubated additional 5 min after addition of Glycine at final concentration of 125 mM, washed 3× with TBS (20 mM Tris-HCl pH 7.6, 150 mM NaCl), pelleted, and frozen in liquid nitrogen^28,61^. Cells were lysed with FastPrep (MP Biomedicals) in lysis buffer (50 mM Hepes-KOH pH 7.5, 140 mM NaCl, 1 mM EDTA, 1% Triton X-100, 0.1% sodium deoxycholate, Roche complete protease inhibitor cocktail) with glass beads, and processed for ChIP using monoclonal anti-myc (9B11) or anti-FLAG (M2 F1804) antibody. ChIP samples were analyzed with dot-blot with ^32^P labeled telomeric DNA probe, scanned with Amersham Typhoon, and quantified with NIH ImageJ software. For sub-telomere ChIP assays, primers indicated in Supplementary Fig 2a and Supplementary Table 4 are used in quantitative PCR with Bio-Rad CFX Connect Real-Time PCR detection system. ChIP sample values were normalized to Input samples and plotted as % precipitated DNA. For cell-cycle-ChIP assays, *cdc25-22* cells were grown in YES liquid culture overnight at 25 °C, shifted to 36 °C for 3 h to arrest cells in G_2_/M-phase, and synchronously released into cell cycle by shifting back to 25 °C, and samples were collected every 20 min to be processed for ChIP assays^28,61^. Raw data values and statistical analysis of ChIP data by two-tailed Student’s *t*-test are shown in Supplementary Data 1.

### Statistics and reproducibility

Statistical analysis and individual raw data values for ChIP experiments are provided as a Microsoft Excel file in Supplementary Data 1. Graphs for these data values, plotted as either bar graphs with mean values plus/minus standard error of mean (SEM) or individual data points are also included in Supplementary Data 1. Pairwise two-tailed student’s *t*-test and one-way ANOVA analysis were performed with Excel. Statistically significant values (*p* < 0.05) are marked with red letters. We utilized multiple independently derived strains in most cases, and multiple independently grown and processed cultures to ensure that all conclusions are fully supported and reproducible. Replicates in experiments were defined as independently cultured and processed samples. For Southern blot, co-IP and western blot analysis, completely independent experiments were repeated at least twice and in many cases more to ensure that all results are reproducible even when only one representative blot is shown for a given experiment.

### Reporting summary

Further information on research design is available in the Nature Research Reporting Summary linked to this article.

## Supplementary information

Supplementary Information

Description of Additional Supplementary Files

Supplementary Data 1

Supplementary Data 2

Reporting Summary

## Data Availability

The authors declare that the data supporting the findings of this study are available within the paper and its Supplementary Information files or upon reasonable request to the corresponding author (T.M.N.).
