# Supplementary material for: Tpz1TPP1 prevents telomerase activation and protects telomeres by modulating the Stn1-Ten1 complex in fission yeast
Source: Commun Biol. 2019 Aug 7;2:297. doi: 10.1038/s42003-019-0546-8 (PMC6686008; doi:10.1038/s42003-019-0546-8)
Supplement: Supplementary file 1 — Supplementary Information [file 42003_2019_546_MOESM1_ESM.pdf]

```
                                                                                75 78
S.pombe      MSNCLKHPWLENGLLNLIKNADVLPIRVFKCQPLQIFEYIRYEHPIRCKLSDTEFYIEAEFSSQSISDLNNFTEKRITSL  80
S.cryophilus MSNILN-PWLESWLLDTMKDTDVLPAHEFKSLPLQLSEYLTYEPSIRAKLSDTRHYIEAEFSPQCIQHLTSFTDKRLTSL  79
S.octosporus MSNILN-PWLESWLLDTLKDADTLPTYEFKSRPLQISEYLTYEPTIHARLSDTRHYIEVEFSPECIQHLSRFTEKRLTSL  79
S.japonicus  MSGLLK-PWLESQLFKKLNDANYIPLKCGQSLPLQIFKFLRFEMPIVALLSDTDYYIEAVFTEESIAAYRKNSEKRLTSL  79

S.pombe      RGGIVTLGNFLIHLIPSQSGIIPWIQVESFNFQGCE-GAVFGNPKAITTSALFNALLQSPYLAALANEFNRSIKEGSSYQ 159
S.cryophilus KGGIFSLGHFSIHLIPQDSYLFPWLYIESFNFFSCE-GVIFGEPKPIVSSPLFRSLLYSPYLLALANELHGCYEHSVLHI 158
S.octosporus KGGIFSLGYFSIHLISQDSYLFPWLYIESFNFFSCE-GVIFGEPKPIISSPLFRSLLCSPYLFALANELNEHYEHSVLYA 158
S.japonicus  RGGIICLRRYFVYLSDVNQVISIRIVVNDFNYLGCEAGALYGDPKRIYASNKLCQLLQSPYLQALIAQAQPSVIVSNSSI 159

S.pombe      EASLSQQEKPNDNTS----------NSRDIKNNIQFHWKNMTSLSIEECIIPKGQQLILEKESEENTTHGIYLEERKMAQ 229
S.cryophilus LREQAVNGDVQKLGTLFLPGS---IEKPKTETSNSFRWKTMTYLDIAQCLIPEGQLRLLETV-DGNTNLDLLESKQIKE- 233
S.octosporus LRKQAINGNVQRLGTLFLPKA---IQKPTKMASDCFRWKTMNSLDLSQCLISDEQSRLLNTE-NANTNIDVCVTKQIEE- 233
S.japonicus  NSDLLLTQSSKKSSAEQSSNASLKISSSSLNSQPAFLWKTMTNLKLEDCLVPKEQQCLLRK--KKSWYPSICDDLSSEET 237

                            242
                                        SWSSS
S.pombe      GLHNSVSETPE---VKQEDNDEDLDAYSWSSSTDSAG--EIPSLPT-NRKILEKIAEKPPPFESPLEDDETPDQTNEHEA 303
S.cryophilus -------NLPDDIHVKEEEQMEI---YSWSSSPEPSA--NVPQLTLFDQQDLQRLAEAEPVSASTISENELISKIDQQKT 301
S.octosporus -------NSPDNTQIKEEEGVEF---YSWSSSPNLNT--NIPLLTHLDQQDLERLAEVEPVSASVLSENELIRKIDQQKK 301
S.japonicus  DSEPPAVEDEDVPVIKEEPDVSAAD-FSWSSSPDTPAKDQVKALRSVNADILNEALRKQPQSPVSYAG----SHASESPT 312
                           Stn1 BINDING

S.pombe      NQVNVSQLPLNPRGSGI--SGRPVESTEQLNSSLTIERSQSIQSTDSKQRV---------ETQSHRRSKIEIFDAQD---- 369
S.cryophilus NYPSVS-VP----GPGIEEPSRVLSSVESHHEEMGQKQDQQEAPTQQAAKLSEVDTLADENKPKQRDNIQLFDAQDDSEA 376
S.octosporus PNDTTTSIS----QPEI-DFSRIVSSVETQRERSGHIQDQQGTPIQRFVEIPDGHISDGESKSKPPDTIELFDAQEDSED 376
S.japonicus  QFPATT-------------FSQHVSNRLVPIAASTQLSAPQ--PVQSQSDLPPI---------LPPSTIPV--------- 359

S.pombe      ---ELFDRSICTTIDDSTGKLLNAEETPIKTGDLHSTSASSVISCTPPAINFTS-------DICNEQIELEYKRKPIPDY 439
S.cryophilus DLEEVDELKLFASLEG--GKTEKSHEARHANQETLKV-ENPVITNTP--FSFEK-------NEDISQHELRFIRRPFPNY 444
S.octosporus DLAEVEELEIFPSLYN--QKTERSHETRNINPKFSKEAENSIITNTP--LNFEK-------SLDMSQHELRYLRRPISDY 445
S.japonicus  PFVPTCEQTIPDSIIKNPTHLQIAPPARTEAVSFHNE---SVISETPSKKHIMSAPTYVVPKAPSRSSSITFLRKALPSF 436

                  449                                                            498  501
S.pombe      DFMKGLETTLQELYVEH---------QSKKRRLELFQLTNNHQKNSEACEMCRLGLPHGSFFELLRDWKKIEEFRNKS-- 508
S.cryophilus SFDLGIQNELTTMYEDYLKENEQRRKRTKRRRSQLFTETPVERSRSE-----------NAFLTFYQDWMRIARHRTDKGK 513
S.octosporus NFDLGIQNELTTIYEDYVKENE-RLRRVKKRRSQPSAQTSLEKTDSE-----------NAFLTFYKDWIRIAKHRKEKGN 513
S.japonicus  DIDTQVQHALHEVYRDYCTGA-----CNKKQLSSISNCKTLVKTKQQLMDIC-----EGEFHDLHKNWLRIKKYRERMLA 506
             Ccq1 BINDING                                                   Poz1 BINDING

S.pombe      --------- 508
S.cryophilus KLDLKK--- 519
S.octosporus IKIQSKKEG 522
S.japonicus  KV------- 508
```

Supplementary Figure 1. Alignment of full length Tpz1 among four *Schizosaccharomyces* species. Identical residues conserved among all four species are marked black, while amino acid residues that maintain similar chemical properties among all four species are marked gray. Regions/Residues important for mediating Tpz1-Stn1, Tpz1-Ccq1, and Tpz1-Poz1 interactions are also indicated.

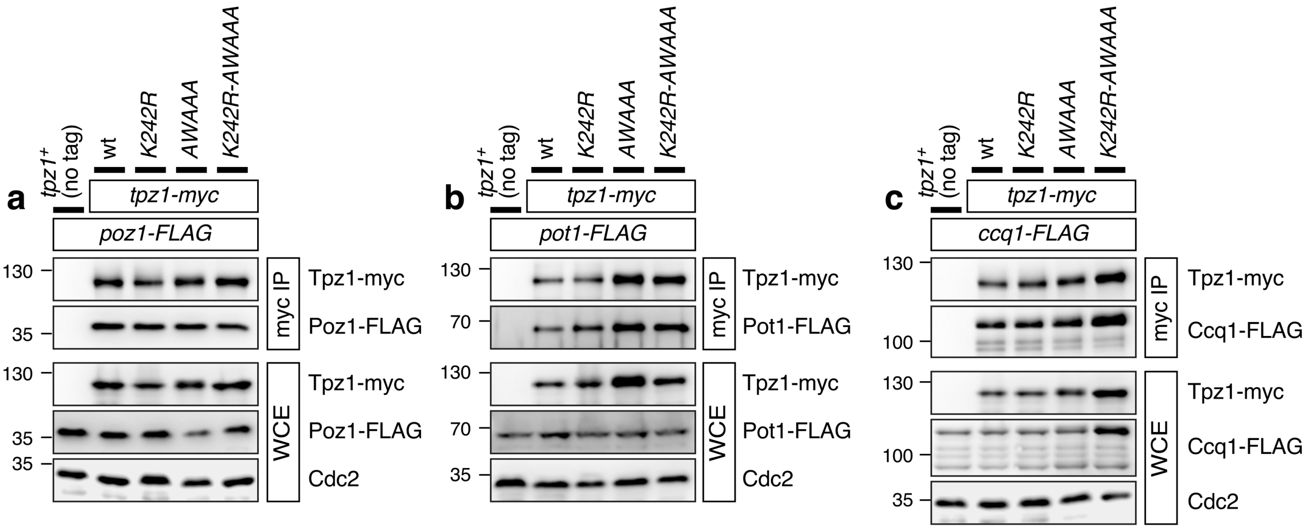

Supplementary Figure 2. Neither *tpz1-K242R* nor *tpz1-AWAAA* mutation interferes with the formation of the shelterin complex. **(a-c)** Co-IP analysis for (a) Tpz1-Poz1, (b) Tpz1-Pot1, and (c) Tpz1-Ccq1 interaction for wild-type (wt), *K242R*, *AWAAA* or *K242R-AWAAA* mutants of Tpz1. Cdc2 western blot served as loading control for whole cell extract (WCE). Molecular weight (kDa) of size markers are indicated.

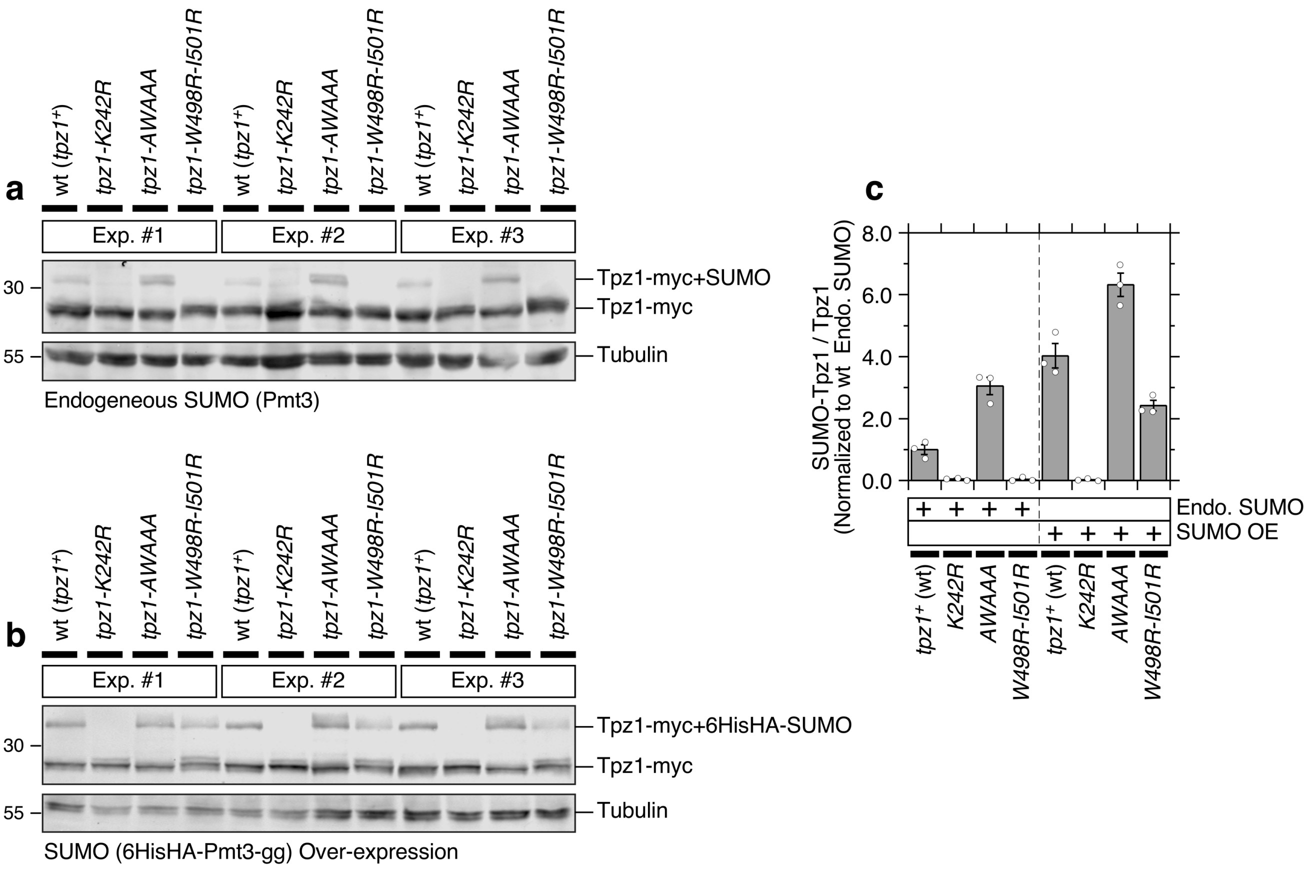

Supplementary Figure 3. Analysis of Tpz1 SUMOylation levels by western blot. Detection of Tpz1 SUMOylation in wild-type (wt) and indicated mutant alleles of Tpz1 with **(a)** endogenous SUMO or **(b)** over-expressed 6HisHA-SUMO. Molecular weight (kDa) of size markers are indicated. (**c**) Quantification of SUMO-Tpz1/Tpz1 ratio, normalized to wild-type cells with endogenous SUMO expression. Error bars represent SEM (n=3). Quantification data is included in Supplementary Data 1.

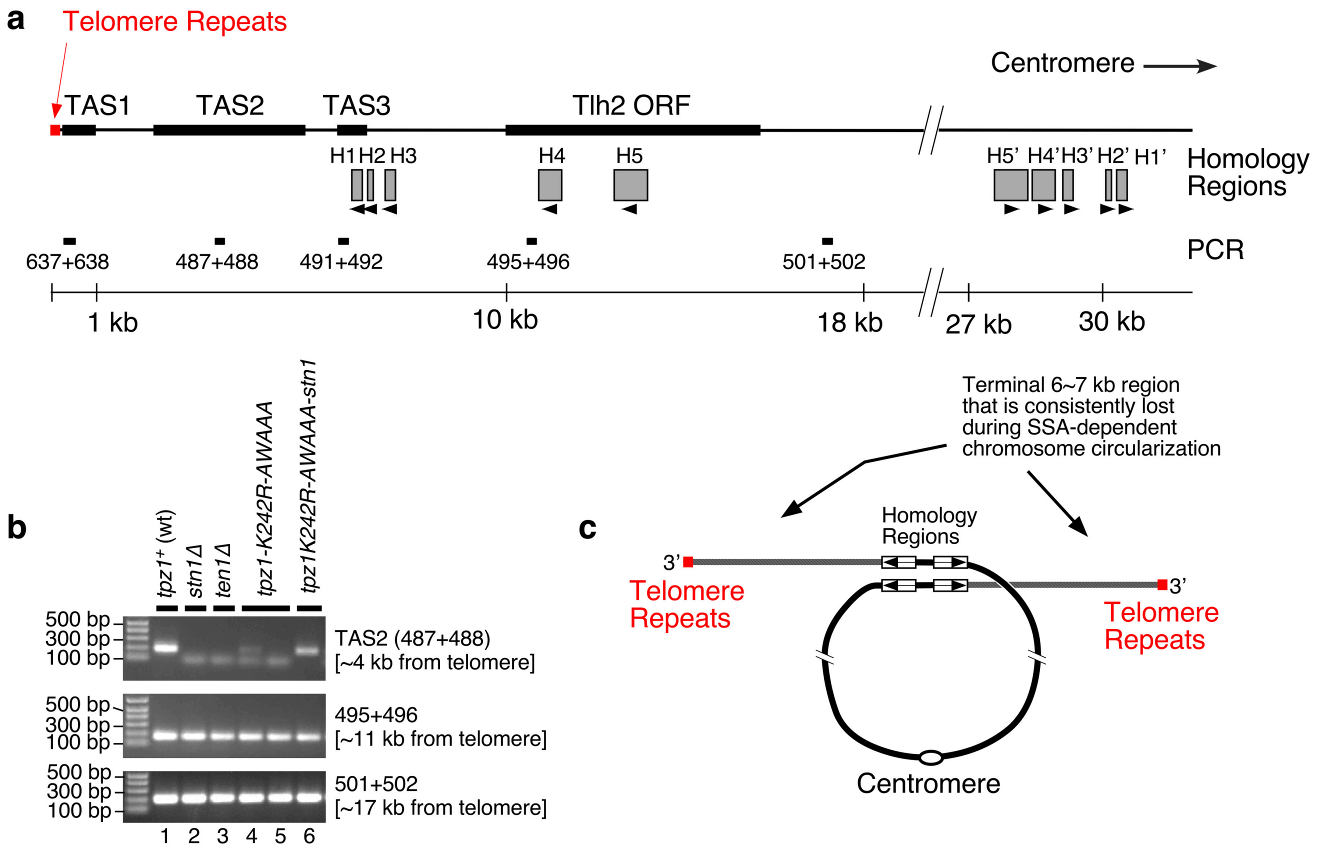

Supplementary Figure 4. Fission yeast strains carrying circular chromosomes lose sub-telomeric sequene due to SSA. **(a)** A schematic map of telomere and sub-telomere regions. Main regions of sub-telomeres[1] and previously identified homology regions utilized in generating circular chromosomes by SSA mechanism[2] are indicated. Locations and primer numbers for PCR products used to characterize sub-telomere regions in (b) or sub-telomere ChIP analysis shown in Supplementary Figure 7 are also indicated. **(b)** PCR analysis of indicated strains for sub-telomere regions as indicated. **(c)** A schematic drawing depicting loss of distal sub-telomere regions after SSA-based fusions that generate circular chromosomes in fission yeast cells defective in telomere protection.

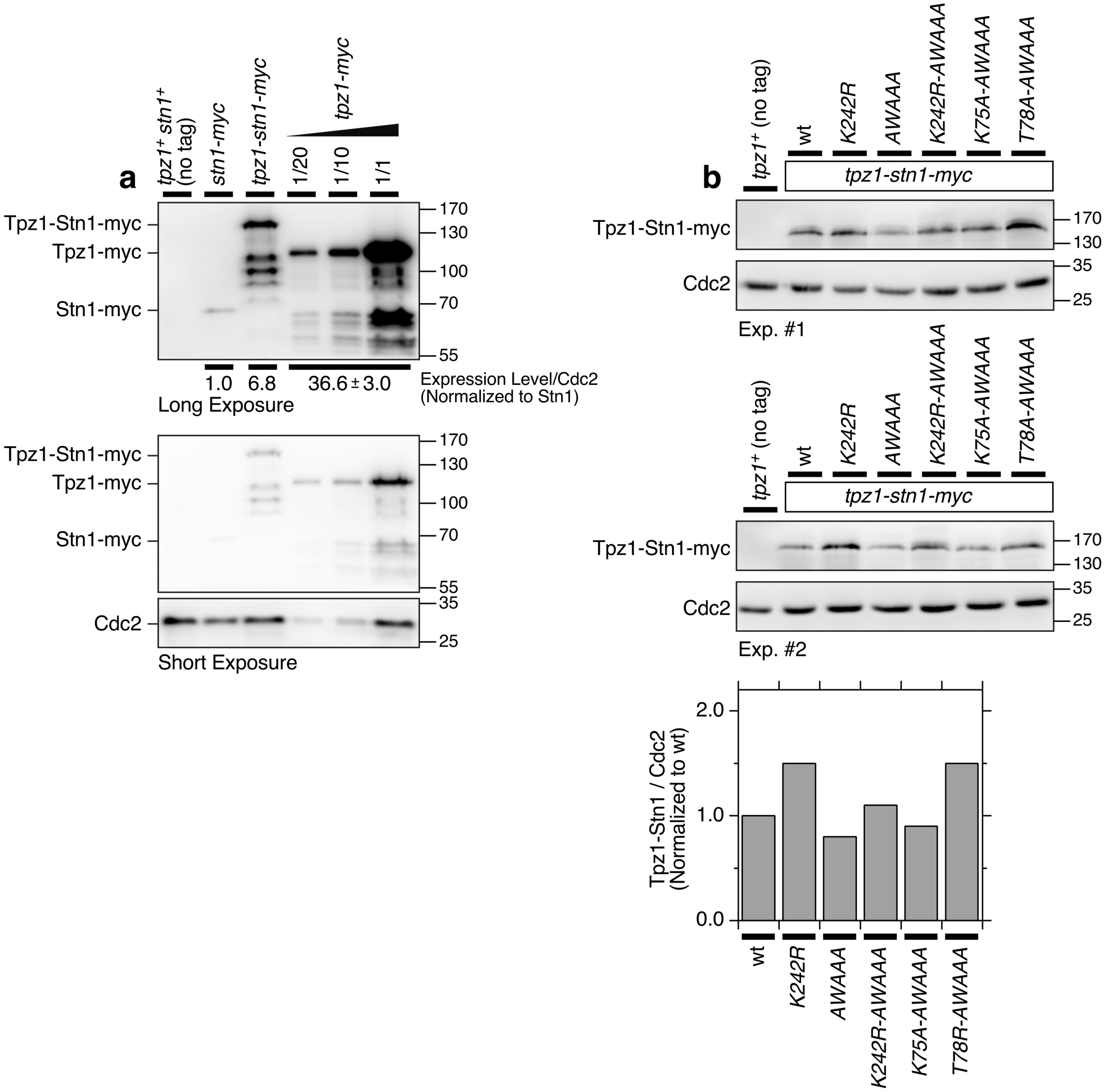

Supplementary Figure 5. Western blot analysis of Stn1, Tpz1 and Tpz1-Stn1 proteins. **(a)** Expression levels for myc-tagged Stn1, Tpz1-Stn1 and Tpz1 were analyzed by western blot. Molecular weight (kDa) of size markers are indicated. Expression levels of indicated proteins were quantified against Cdc2 loading control, and normalized to the value obtained for Stn1. For Tpz1, average of Tpz1/Cdc2 ratio among three indicated dilutions and SEM is indicated. **(b)** Comparison of Tpz1-Stn1 expression levels among wild-type (wt) and indicated tpz1 mutant alleles. Western blot results from two independent experiments are shown. Average of Tpz1-Stn1/Cdc2 ratio from two experiments shown (normalized to wt) were plotted. No major changes in expression levels for Tpz1-Stn1 fusion protein were observed among different mutants. Quantification data is included in Supplementary Data 1

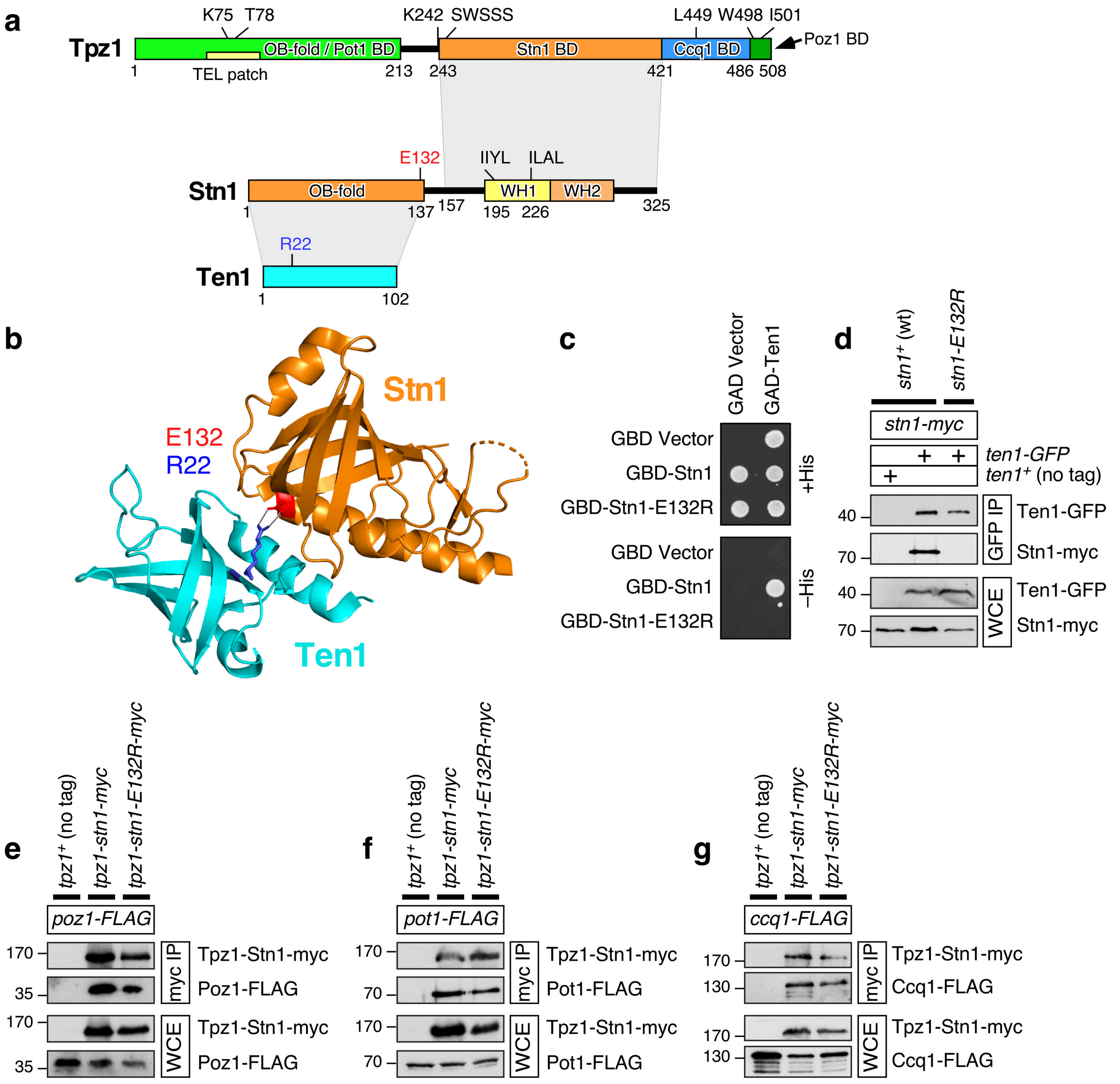

Supplementary Figure 6. Additional characterization of *stn1-E132R* mutation and Tpz1-Stn1 fusion construct. **(a)** A schematic representation of Tpz1, Stn1 and Ten1. Domains previously found to mediate Tpz1-Stn1 and Stn1-Ten1 interaction are indicated with gray shaded areas[3,4]. In addition, residues that are important for mediating Stn1-Ten1 interaction (Stn1 Glu132 and Ten1 Arg22) and possible SUMO-Interacting Motif (SIM) sequences in Stn1 (IIYL and ILAL) are indicated. For Tpz1, see Fig 1b legend for details on indicated domains and residues. **(b)** Crystal structure of Stn1-Ten1 complex[5]. Amino acid residues for Stn1 Ser16-His154 and Ten1 Asp2-Asp102 are shown (PDF ID: 3KF6). Side chains for Stn1 Glu132 and Ten1 Arg22, predicted to play a critical role in facilitating Stn1-Ten1 interaction are shown, and highlighted in red and blue, respectively. **(c, d)** *Stn1-E132R* mutation disrupts Stn1-Ten1 interaction as detected by (c) Y2H assay and (d) co-IP. **(e-g)** Based on co-IP assays, both Tpz1-Stn1 and Tpz1-Stn1-E132R fusion proteins can interact with the other shelterin subunits (e) Poz1, (f) Pot1 and (g) Ccq1. Molecular weight (kDa) of size markers are indicated.

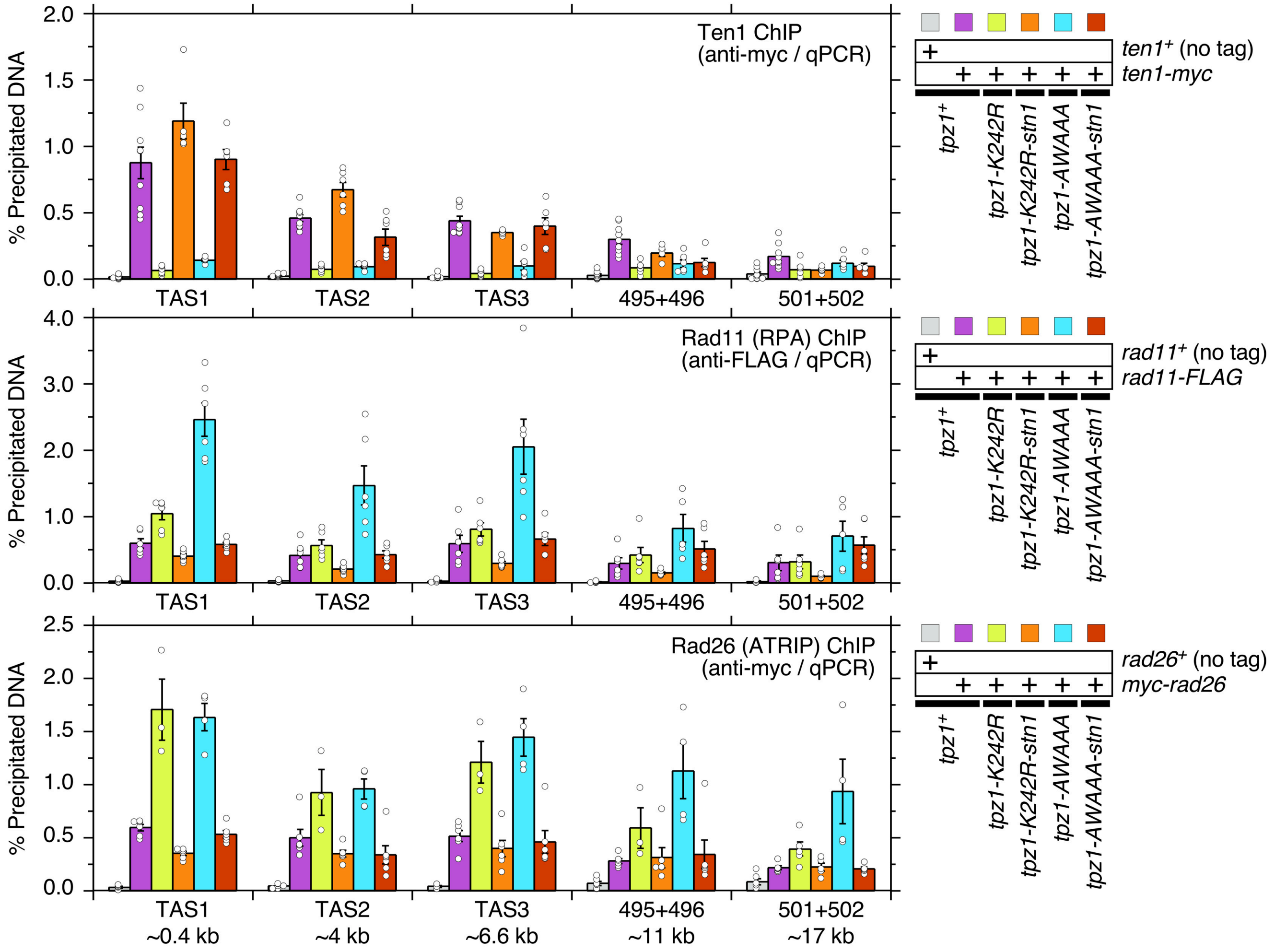

Supplementary Figure 7. ChIP analysis of sub-telomere regions for Ten1, Rad11 and Rad26. Quantitative PCR-based ChIP analysis for indicated genetic backgrounds are carried out for Ten1, Rad11 (RPA) and Rad26^{ATRIP}. See supplementary Fig 4a for locations of PCR products within sub-telomere regions, used in ChIP assays. Plots show mean values plus/minus SEM and distribution of individual data points from at least 3 independent experiments. Raw data values and statistical analysis of ChIP assays by two-tailed Student's t-test are shown in Supplementary Data 1.

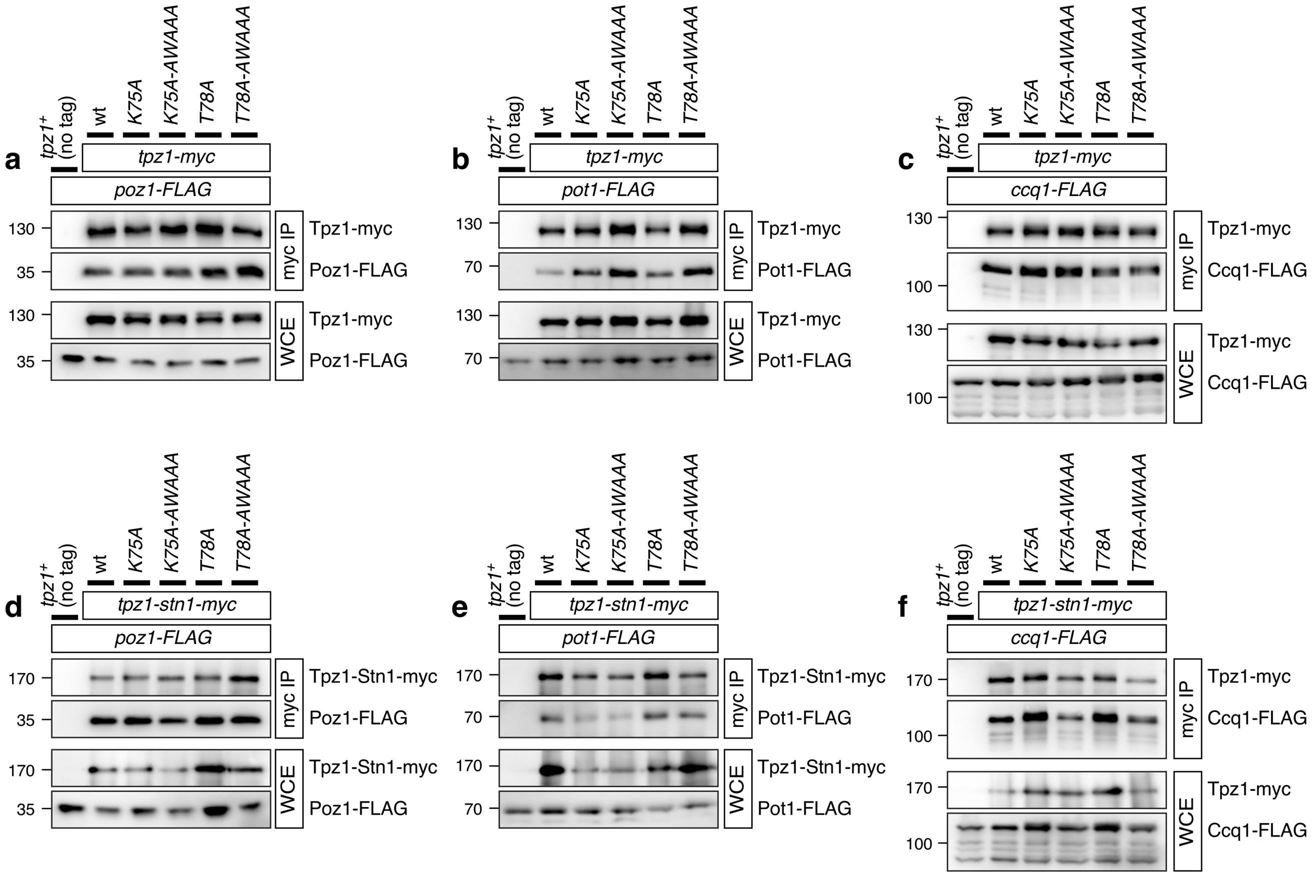

Supplementary Figure 8. Mutant constructs of Tpz1 or Tpz1-Stn1 can still form the shelterin complex. **(a-c)** Co-IP analysis for (a) Tpz1-Poz1, (b) Tpz1-Pot1, and (c) Tpz1-Ccq1 interaction for wild-type (wt), and indicated mutants of Tpz1. **(d-f)** Co-IP analysis for (d) Tpz1-Poz1, (e) Tpz1-Pot1, and (f) Tpz1-Ccq1 interaction for wild-type (wt), and indicated Tpz1 mutant versions of Tpz1-Stn1 fusion protein. Molecular weight (kDa) of size markers are indicated.

Fig 2c

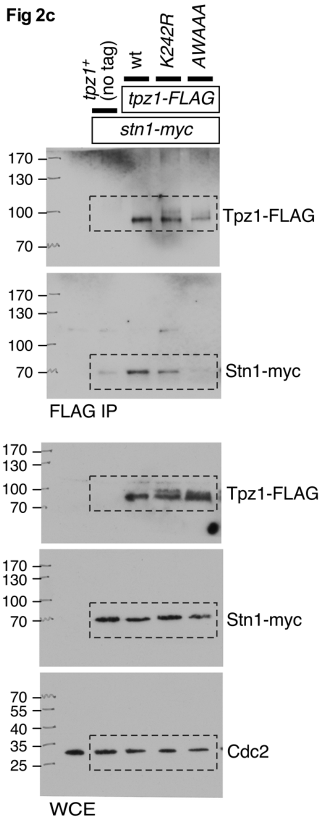

Fig 3a

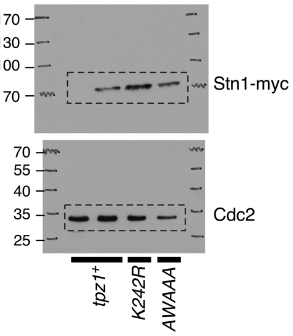

Fig 3b

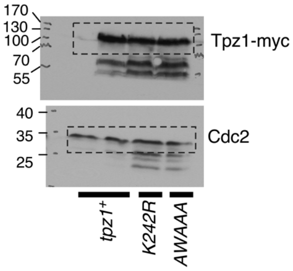

Fig 3d

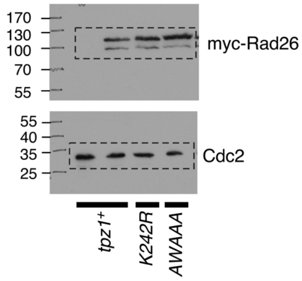

Fig 3c

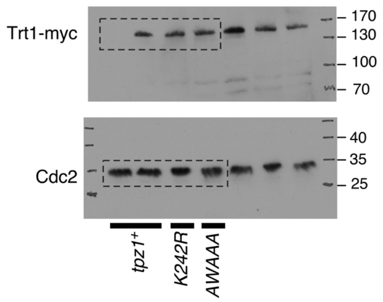

Fig 5a

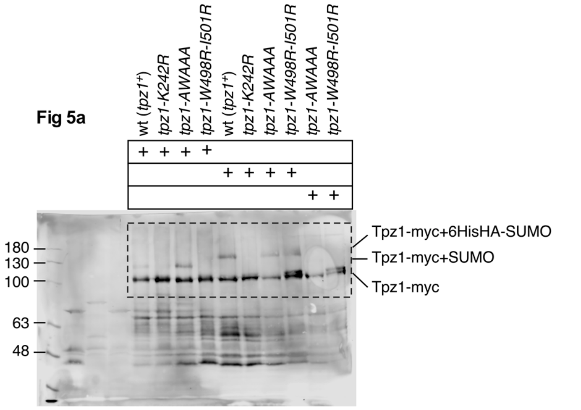

Fig 6e

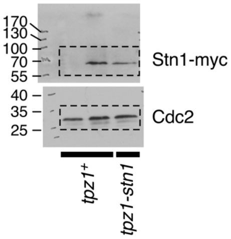

Fig 6f

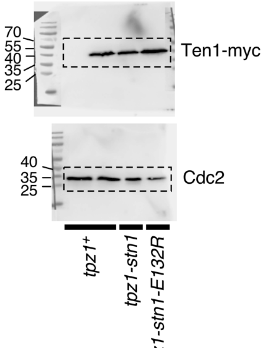

Fig 8a

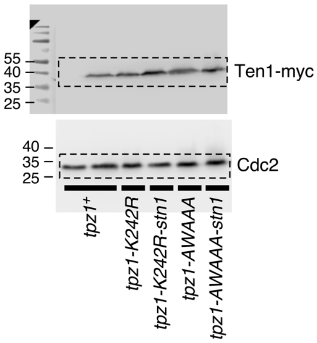

Fig 8b

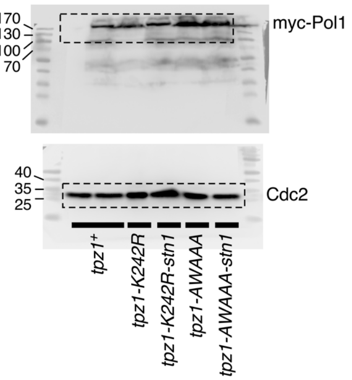

Fig 8c

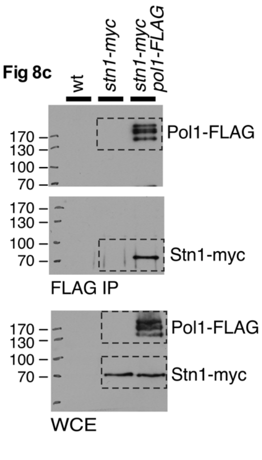

Fig 8d

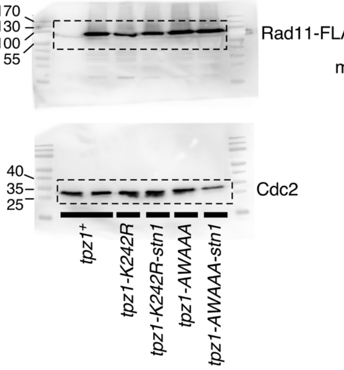

Fig 8e

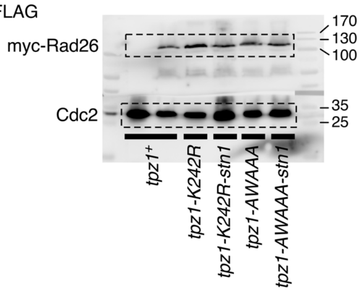

Fig 8f

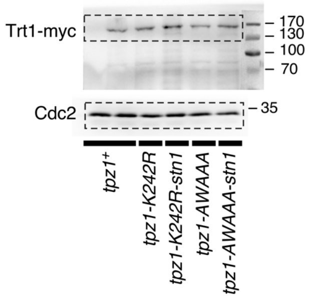

Fig 9d

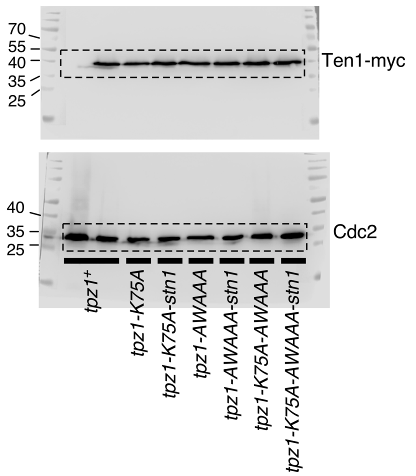

Fig 9e

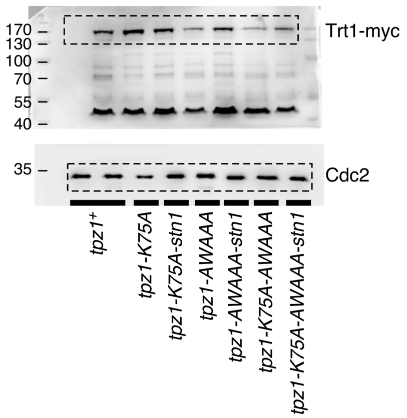

Supp Fig 2a

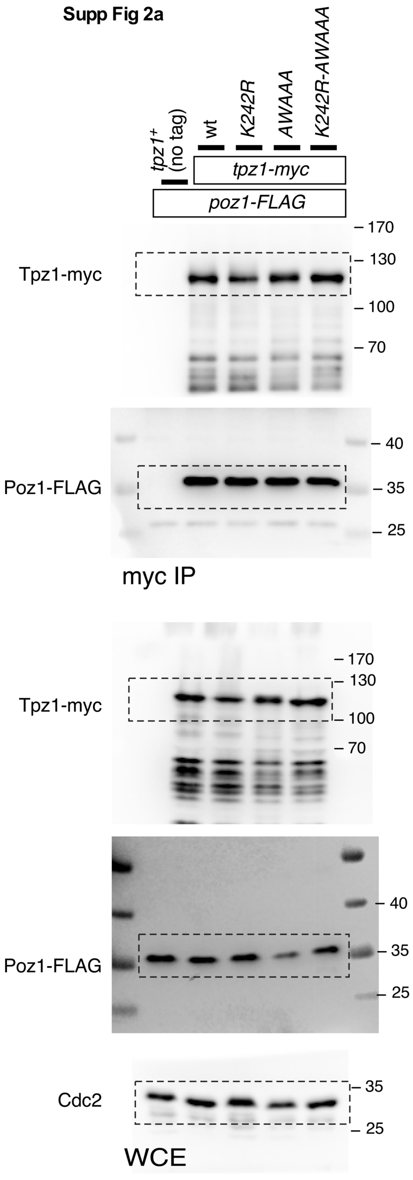

Supp Fig 2b

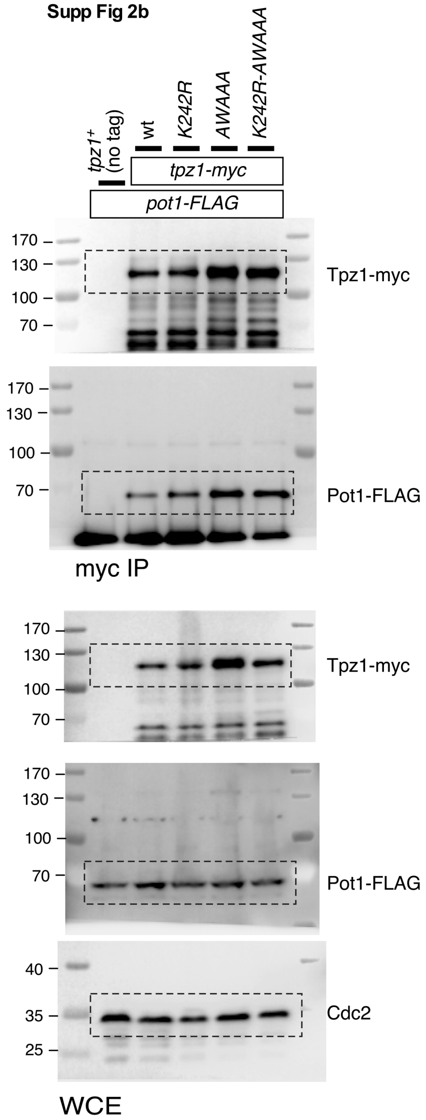

Supp Fig 2c

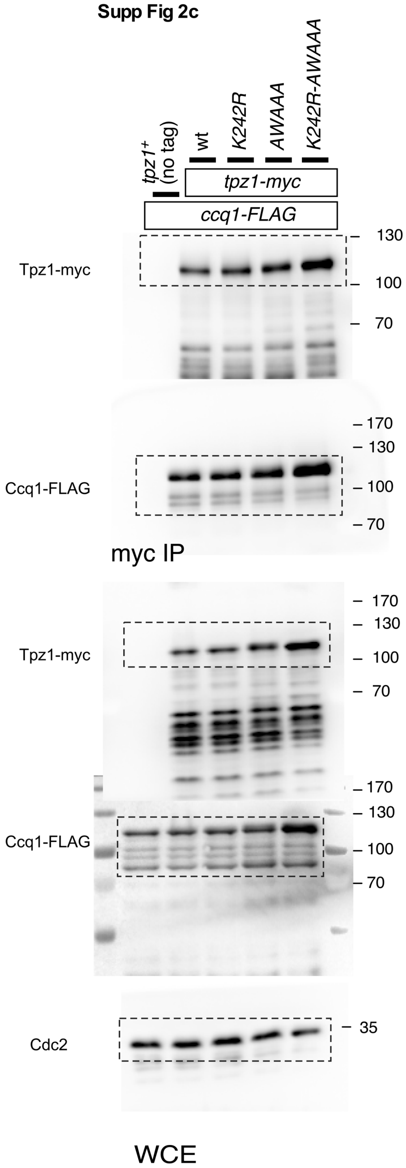

Supp Fig 3a

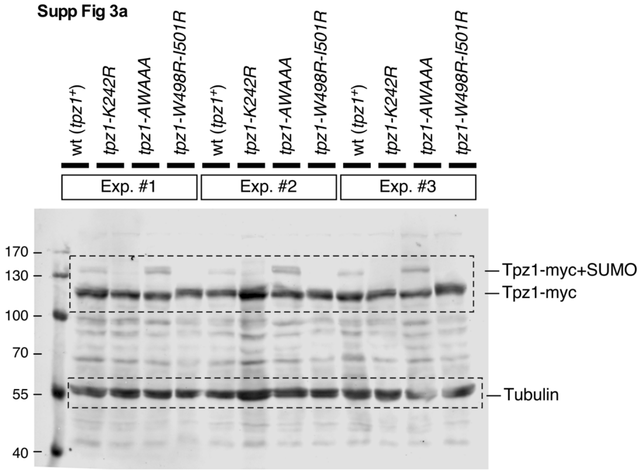

Endogeneous SUMO (Pmt3)

Supp Fig 3b

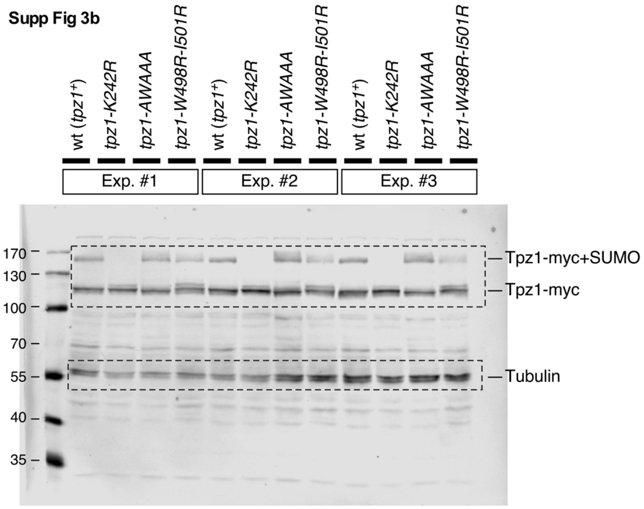

SUMO (6HisHA-Pmt3-gg) Over-expression

Supplementary Fig 5a

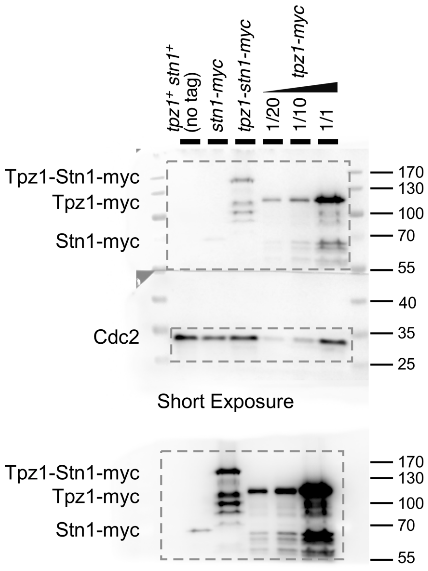

Long Exposure

Supplementary Fig 5b

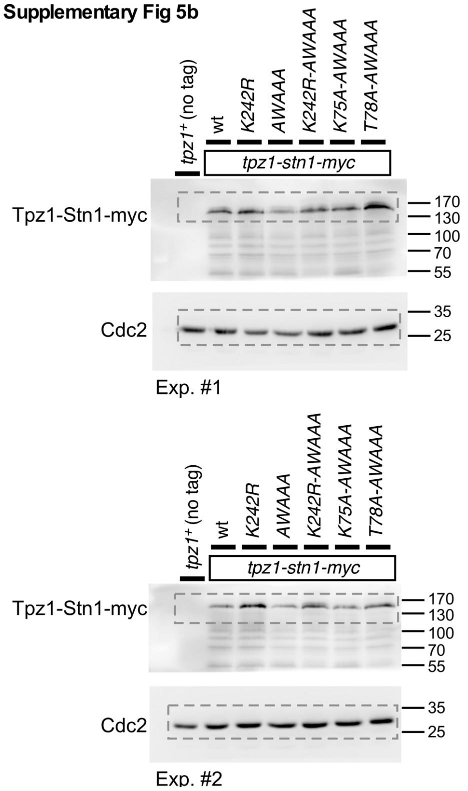

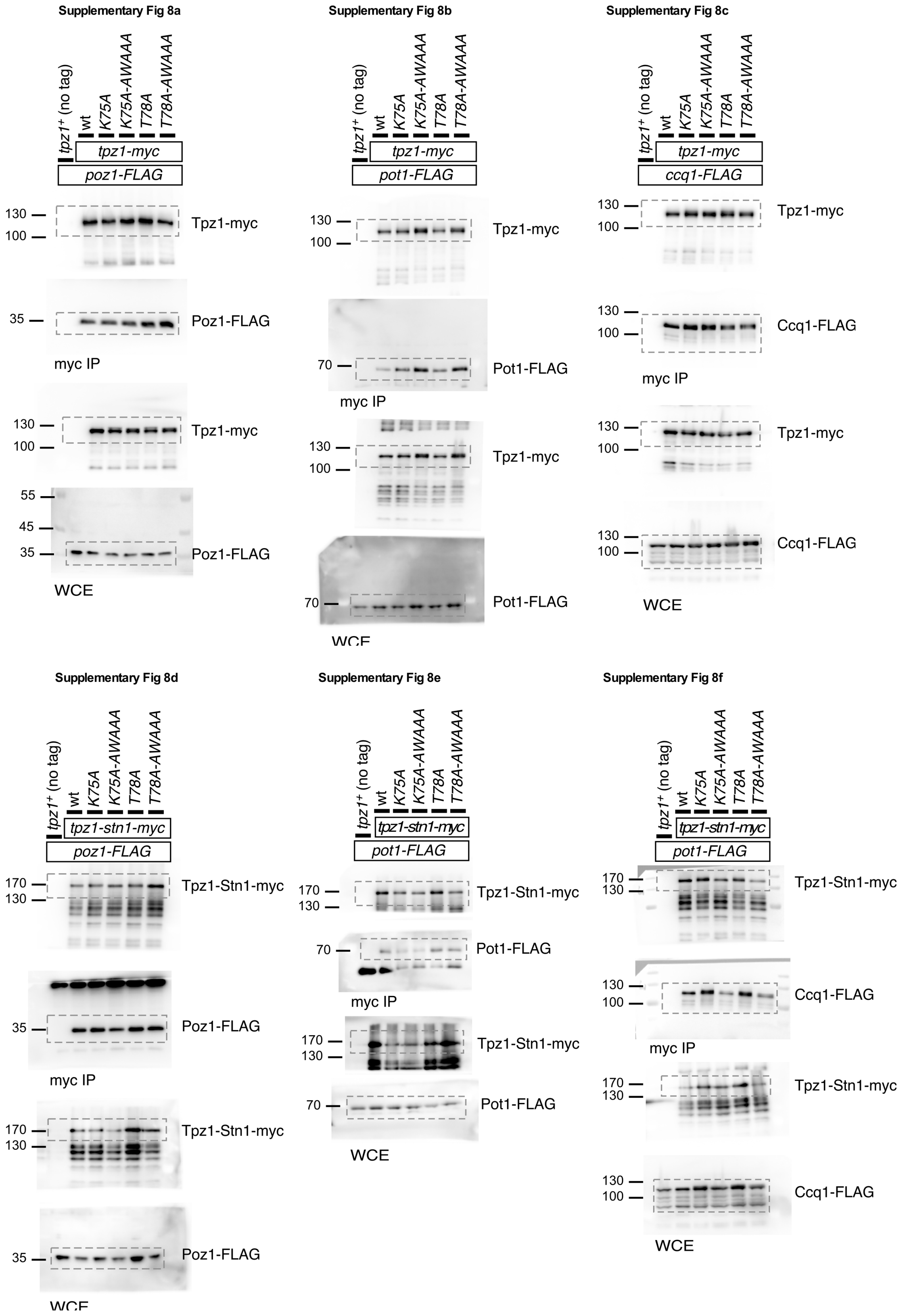

Supplementary Figure 9. Uncropped western blot gels for indicated figures. Areas of gels shown in final figures are marked with dashed boxes. Sizes of protein molecular weight markers (kDa) are also indicated.

SUPPLEMENTARY REFERENCES

1. Harland, J.L., Chang, Y.T., Moser, B.A. & Nakamura, T.M. Tpz1-Ccq1 and Tpz1-Poz1 interactions within fission yeast Shelterin modulate Ccq1 Thr93 phosphorylation and telomerase recruitment. *PLoS Genet* **10**, e1004708 (2014).
2. Wang, X. & Baumann, P. Chromosome fusions following telomere loss are mediated by single-strand annealing. *Mol Cell* **31**, 463-73 (2008).
3. Chang, Y.T., Moser, B.A. & Nakamura, T.M. Fission yeast shelterin regulates DNA Polymerases and $Rad3^{ATR}$ kinase to limit telomere extension. *PLoS Genet* **9**, e1003936 (2013).
4. Matmati, S. et al. The fission yeast Stn1-Ten1 complex limits telomerase activity via its SUMO-interacting motif and promotes telomeres replication. *Sci Adv* **4**, eaar2740 (2018).
5. Sun, J. et al. Stn1-Ten1 is an Rpa2-Rpa3-like complex at telomeres. *Genes Dev* **23**, 2900-14 (2009).
