# Supplementary material for: Tpz1TPP1 prevents telomerase activation and protects telomeres by modulating the Stn1-Ten1 complex in fission yeast
Source: Commun Biol. 2019 Aug 7;2:297. doi: 10.1038/s42003-019-0546-8 (PMC6686008; doi:10.1038/s42003-019-0546-8)
Supplement: Supplementary file 2 — Description of Additional Supplementary Files [file 42003_2019_546_MOESM2_ESM.docx]

**Description of Additional Supplementary Files**

File Name: Supplementary Data 1

Description: Raw data and statistical analysis.

File Name: Supplementary Data 2

Description: 4 tables containing:

1. Fission yeast strains used in this study.
2. Source of various mutated and tagged alleles for fission yeast strains used in this study.
3. Plasmids used in this study.
4. DNA primers used in this study.
