# Supplementary material for: Tpz1TPP1 prevents telomerase activation and protects telomeres by modulating the Stn1-Ten1 complex in fission yeast
Source: Commun Biol. 2019 Aug 7;2:297. doi: 10.1038/s42003-019-0546-8 (PMC6686008; doi:10.1038/s42003-019-0546-8)
Supplement: Supplementary file 5 — Reporting Summary [file 42003_2019_546_MOESM5_ESM.pdf]

nature research

Corresponding author(s): Toru Nakamura

Reporting Summary

Nature Research wishes to improve the reproducibility of the work that we publish. This form provides structure for consistency and transparency in reporting. For further information on Nature Research policies, see Authors & Referees and the Editorial Policy Checklist.

Statistical parameters

When statistical analyses are reported, confirm that the following items are present in the relevant location (e.g. figure legend, table legend, main text, or Methods section).

n/a	Confirmed	
☐	☒	The exact sample size (*n*) for each experimental group/condition, given as a discrete number and unit of measurement
☐	☒	An indication of whether measurements were taken from distinct samples or whether the same sample was measured repeatedly
☐	☒	The statistical test(s) used AND whether they are one- or two-sided<br>*Only common tests should be described solely by name; describe more complex techniques in the Methods section.*
☒	☐	A description of all covariates tested
☒	☐	A description of any assumptions or corrections, such as tests of normality and adjustment for multiple comparisons
☐	☒	A full description of the statistics including central tendency (e.g. means) or other basic estimates (e.g. regression coefficient) AND variation (e.g. standard deviation) or associated estimates of uncertainty (e.g. confidence intervals)
☐	☒	For null hypothesis testing, the test statistic (e.g. *F*, *t*, *r*) with confidence intervals, effect sizes, degrees of freedom and *P* value noted<br>*Give P values as exact values whenever suitable.*
☒	☐	For Bayesian analysis, information on the choice of priors and Markov chain Monte Carlo settings
☒	☐	For hierarchical and complex designs, identification of the appropriate level for tests and full reporting of outcomes
☒	☐	Estimates of effect sizes (e.g. Cohen's *d*, Pearson's *r*), indicating how they were calculated
☐	☒	Clearly defined error bars<br>*State explicitly what error bars represent (e.g. SD, SE, CI)*

Our web collection on statistics for biologists may be useful.

Software and code

Policy information about availability of computer code

Data collection	Dot blot ChIP assays were scanned with Phosphor-imager and quantified with ImageJ. Realtime PCR data was collected with BioRad software, and exported to Microsoft Excel.
Data analysis	Microsoft Excel. Western blots were quantified with LI-COR Image Studio software.

For manuscripts utilizing custom algorithms or software that are central to the research but not yet described in published literature, software must be made available to editors/reviewers upon request. We strongly encourage code deposition in a community repository (e.g. GitHub). See the Nature Research guidelines for submitting code & software for further information.

Data

Policy information about availability of data

All manuscripts must include a data availability statement. This statement should provide the following information, where applicable:
- Accession codes, unique identifiers, or web links for publicly available datasets
- A list of figures that have associated raw data
- A description of any restrictions on data availability

Raw data values are provided in Supplementary Dataset

Field-specific reporting

Please select the best fit for your research. If you are not sure, read the appropriate sections before making your selection.

☒ Life sciences ☐ Behavioural & social sciences ☐ Ecological, evolutionary & environmental sciences

For a reference copy of the document with all sections, see nature.com/authors/policies/ReportingSummary-flat.pdf

Life sciences study design

All studies must disclose on these points even when the disclosure is negative.

Sample size	We utilized multiple independently derived strains in most cases and multiple independently grown cultures to ensure all conclusions are fully supported. Since we perform all our assays with yeast cultures that contain millions of cells, and do not evaluate data from a single cell, it is not entirely clear if the issue of "sample size" is really relevant here.
Data exclusions	Data was excluded only in cases where experiments clearly failed (i.e. loss of sample, inhibition in qPCR, etc.)
Replication	Experiments have been extensively repeated to ensure reproducibility.
Randomization	Samples were not randomized, but multiple independent strains were used in most cases to ensure results were reproducible.
Blinding	Not applicable.

Reporting for specific materials, systems and methods

Materials & experimental systems

n/a	Involved in the study
☐	☒ Unique biological materials
☐	☒ Antibodies
☒	☐ Eukaryotic cell lines
☒	☐ Palaeontology
☒	☐ Animals and other organisms
☒	☐ Human research participants

Methods

n/a	Involved in the study
☒	☐ ChIP-seq
☒	☐ Flow cytometry
☒	☐ MRI-based neuroimaging

Unique biological materials

Policy information about availability of materials

Obtaining unique materials	All reagents described in the manuscript will be freely available to researchers who request them.

Antibodies

Antibodies used	monoclonal anti-FLAG (M2-F1804, Sigma), monoclonal anti-myc (9B11, Cell Signaling), monoclonal anti-GFP (clones 7.1 and 13.1, Roche), monoclonal anti-alpha-tubulin (clone B-5-1-2 T5168, Sigma), monoclonal anti-Cdc2 (y100.4, SCBT), HRP-conjugated (goat) anti-mouse (Pierce, 31430)
Validation	For each antibody against tagged protein, no-tag control strain was included to ensure specificity. Anti-Cdc2 and anti-tubulin have been extensively used by multiple fission yeast labs as loading control in western blot analysis.
